# The gasdermin family: from pyroptosis mechanisms to therapeutic targets

**DOI:** 10.1038/s41392-026-02867-2

**Published:** 2026-10-02

**Authors:** Weilv Xu, Shiyang Liu, Zexu Yu, Jinhuang Shi, Yang Yang, Fushan Shi

**Affiliations:** 1 MOA Key Laboratory of Animal Virology, Zhejiang University Center for Veterinary Sciences, Hangzhou, China; 2 Department of Veterinary Medicine, College of Animal Sciences, Zhejiang University, Hangzhou, China; 3 Key Laboratory of Applied Technology on Green-Eco-Healthy Animal Husbandry of Zhejiang Province, Zhejiang Provincial Engineering Research Center for Animal Health Diagnostics & Advanced Technology, Zhejiang International Science and Technology Cooperation Base for Veterinary Medicine and Health Management, China-Australia Joint Laboratory for Animal Health Big Data Analytics, College of Animal Science and Technology & College of Veterinary Medicine of Zhejiang A&F University, Hangzhou, China; 4 Zhejiang Provincial Engineering Research Center of Animal Biological Products, Zhejiang University Center for Veterinary Sciences, Hangzhou, China

**Keywords:** Inflammation, Innate immunity

## Abstract

The gasdermin (GSDM) family of proteins has emerged as the central executioner of pyroptosis, a form of inflammatory cell death characterized by plasma membrane rupture and the release of pro-inflammatory cytokines such as interleukin-1β (IL-1β), IL-18 and high mobility group protein B1 (HMGB1). Emerging evidence indicates that GSDMs exert diverse functions in health and various disease contexts, which can be either dependent on or independent of pyroptosis, including facilitating mucus secretion, regulating bone resorption, and modulating mitochondrial metabolism. This review provides a comprehensive overview of the GSDM family, detailing the molecular mechanisms of activation, regulation, pore formation, cell rupture and membrane repair. We explored the extensive crosstalk between GSDMs and other cell death modalities, including apoptosis, necroptosis, NETosis, and PANoptosis. Furthermore, we systematically discuss GSDMs’ ‘double-edged sword’ roles in diverse pathologies, ranging from host defense in infectious diseases and chronic inflammation in autoimmune disorders to their complex, context-dependent function in cancer progression and immunity. Finally, we evaluate emerging therapeutic strategies, encompassing small-molecule inhibitors such as disulfiram (DSF), necrosulfonamide (NSA) and dimethyl fumarate (DMF), as well as novel material-based delivery systems. Collectively, these advances underscore the therapeutic potential of targeting GSDMs for precise clinical intervention to treat both inflammatory and non-inflammatory diseases, as well as to enhance cancer immunotherapy.

## Introduction

The Gasdermin (GSDM) family is a group of conserved proteins that are key to pyroptosis, a novel type of inflammatory cell death characterized by cell swelling, plasma membrane rupture, and the release of inflammatory cytokines.^1–6^ Originally identified and named for their specific expression in the gastrointestinal tract and skin, these proteins are now recognized as central players in innate immunity and disease pathology.^7–9^ In humans, the GSDM family is composed of six members: Gasdermin A (GSDMA), Gasdermin B (GSDMB), Gasdermin C (GSDMC), Gasdermin D (GSDMD), Gasdermin E (GSDME, also known as DFNA5), and DFNB59 (Pejvakin/PJVK). These paralogues share approximately 45% sequence homology and cluster on chromosomes 17q21 (GSDMA, GSDMB) and 8q24 (GSDMC, GSDMD), suggesting evolutionary expansion via gene duplication.^10–12^ Most GSDMs, excluding Pejvakin (PJVK), share a two-domain architecture that regulates autoinhibition. These proteins are composed of a cytotoxic N-terminal domain (NT) and a repressor C-terminal domain (CT) connected by a flexible linker.^13,14^ The fundamental mechanism of GSDM activation involves the proteolytic cleavage of this linker by specific enzymes, ranging from inflammatory caspases to granzymes and bacterial proteases.^13–25^ This cleavage releases GSDM-NT to translocate to the plasma membrane and assemble into transmembrane pores.^26,27^ These pores disrupt cellular homeostasis, driving osmotic lysis and inflammatory bursts.^20,28–31^

While GSDMs are best known for their roles in pyroptosis, emerging evidence reveals that they also have crucial non-pyroptotic functions in both physiological and pathological contexts.^1,7,32–35^ Beyond mediating pyroptosis, GSDMs can regulate diverse biological processes in living cells, from modulating mitochondrial homeostasis and gene expression to influencing cell proliferation and differentiation.^32,36–39^ Consequently, their dysregulation is now investigated in a broader spectrum of pathologies than previously recognized, extending beyond septic shock and inflammation to include autoimmune disorders and cancer progression.^40–44^ This review aims to offer a comprehensive overview of GSDMs, summarizing the mechanisms of pyroptosis, their emerging non-pyroptotic functions in physiological and pathological states, and strategies to target gasdermins for therapeutic intervention.

## Mechanisms of pyroptosis

While pyroptosis was originally coined in 2001, the identification of GSDMs as direct executioners in 2015 established its distinct molecular definition.^45^ GSDMs are typically activated by proteolytic cleavage, although specific mutations or post-translational modifications have also been reported to induce activation.^13,31,46–49^ Apart from cleavage that induces pyroptosis, emerging evidence indicates that GSDMs can be cleaved at multiple sites, liberating fragments with different biological functions.^19,50,51^ These findings are summarized in Table 1 and are detailed further in the subsequent section, “Gasdermins’ non-pyroptotic function in health and various disease contexts”. Active GSDM-NT rapidly translocates to the plasma membrane, binding to inner leaflet lipids and oligomerizing into large, ring-shaped transmembrane pores with inner diameters of approximately 15 to 21.5 nm.^26^ These pores disrupt cellular homeostasis by dissipating ionic gradients, causing a massive influx of water that drives cell swelling and eventual osmotic lysis.^27^ This rupture releases intracellular contents, including diverse DAMPs and mature pro-inflammatory cytokines such as IL-1β and IL-18.^52^ This section provides an overview of pyroptosis, detailing the activation pathways of GSDMs, the mechanisms of pore formation and the downstream consequences, including cell rupture and membrane repair.

**Table 1:** Enzymatic cleavage of gasdermins and the functions of the resulting fragments

GSDM member	Gene and chromosomal location	Protein cleavage enzymes and sites	Biological functions of cleavage fragments	Refs
GSDMA	Human: GSDMA (17q21.1) Mouse: Gsdma1-3 (11D)	SpeB (Q246 human)	Pyroptosis (NT)	^16,17^
		Caspase-1 (nonmammals) (not known)		^139^
		Caspase-3/4 (D75, D241 porcine)	Inhibits pyroptosis	^140^
GSDMB	Human: GSDMB (17q21.1) Mouse: None	GZMA (Major-K244, Minor-K229 human)	Pyroptosis (NT)	^25^
		Caspase-1 (D236 human)	Pyroptosis (NT)	^148^
		Caspase-3/6/7 (D237 porcine)	Pyroptosis (NT)	^147^
		Caspase-7 (D91 human)	Inhibits pyroptosis (92–417 aa)	^19^
GSDMC	Human: GSDMC (8q24.21) Mouse: Gsdmc1-4 (15D1)	Caspase-6/8 (D365 human)	Pyroptosis (NT)	^150^
		Caspase-8 (D240 human)	Pyroptosis (NT)	^151^
		GZMB (D276 human)	Pyroptosis (NT)	^153^
		CTSS (multiple sites)	Amplifies anti-helminth immunity (NT)	^447^
GSDMD	Human: GSDMD (8q24.3) Mouse: Gsdmd (15D3-E1)	Caspase-1/4/5/11 (D275 human; D276 mouse)	Pyroptosis (NT); Inhibits the type-I interferon response (CT)	^13,14^
		Caspase-3/8 (D88, D276 mouse)	Inhibits bone resorption (NT-p20)	^51^
		Caspase-3/7 (D87 human; D88 mouse)	Maintains food tolerance (NT-p13)	^50^
		Not known	Promotes host resistance to *Leishmania* infection (NT-p25)	^133^
		Not known (R290 human; R311 mouse)	Promotes type-Ⅱ immunity (NT-p35 human, NT-p40 mouse)	^134^
GSDME	Human: GSDME (7p15.3) Mouse: Gsdme (6B2.3)	Caspase-3 (D270 human)	Pyroptosis (NT)	^23^
		GZMB (D270 human)	Pyroptosis (NT)	^24^
DFNB59	Human: DFNB59 (2q31.2) Mouse: Dfnb59 (2C3)	Not known	Not known	/

### Autoinhibition structure

Full-length GSDMs are autoinhibited through an extensive interface driven by electrostatic and hydrophobic forces. Mechanistically, the acidic GSDM-CT domain masks the positively charged β1-β2 loop and α1-helix of the GSDM-NT domain, thereby preventing activation.^31,46,47,53,54^ In GSDMA3 and GSDMB, an additional autoinhibitory interface between the α4-helix in the GSDM-NT domain and the hydrophobic groove in the GSDM-CT domain reinforces autoinhibition.^53,54^

### GSDM activation

#### GSDMD

The canonical pyroptosis pathway is defined by inflammasome-mediated activation of GSDMD.^15,20^ The process generally begins when pattern recognition receptors (PRRs) detect pathogen-associated molecular patterns (PAMPs) or damage-associated molecular patterns (DAMPs).^55,56^ Numerous inflammasomes have been identified, including NLRP3, NLRP1, AIM2, and NLRC4.^55,57,58^ The most common trigger for the NLRP3 inflammasome is potassium efflux, which can be caused by ATP binding to P2X7 receptors or bacterial pore-forming toxins such as nigericin and maitotoxin.^59–62^ Other triggers include calcium influx, lysosomal disruption (releasing cathepsin B) and mitochondrial dysfunction (releasing oxidized mtDNA and ROS).^63–68^ Unlike NLRP3, NLRP1 and CARD8 activation rely on ‘functional degradation’, where proteasomal destruction of their N-terminal fragments releases bioactive C-terminal domains to initiate assembly.^69–81^ NAIP-NLRC4 (bacterial flagellin/T3SS) and AIM2 (cytosolic dsDNA) are driven by direct ligand binding, while Pyrin indirectly detects pathogen activity by sensing RhoA dephosphorylation.^82–89^ Other specialized sensors include NLRP6, which utilizes liquid‒liquid phase separation, and NLRP12, which scaffolds PANoptosomes in response to heme and PAMPs.^90–96^ Additional sensors respond to specific molecular cues, including RNA via the helicase DHX9 (NLRP9), mitochondrial damage (NLRP10), nuclear viral DNA (IFI16), bacterial lipopeptides (NLRP7), and viral nucleoproteins (MxA).^97–102^

**Fig. 1:**
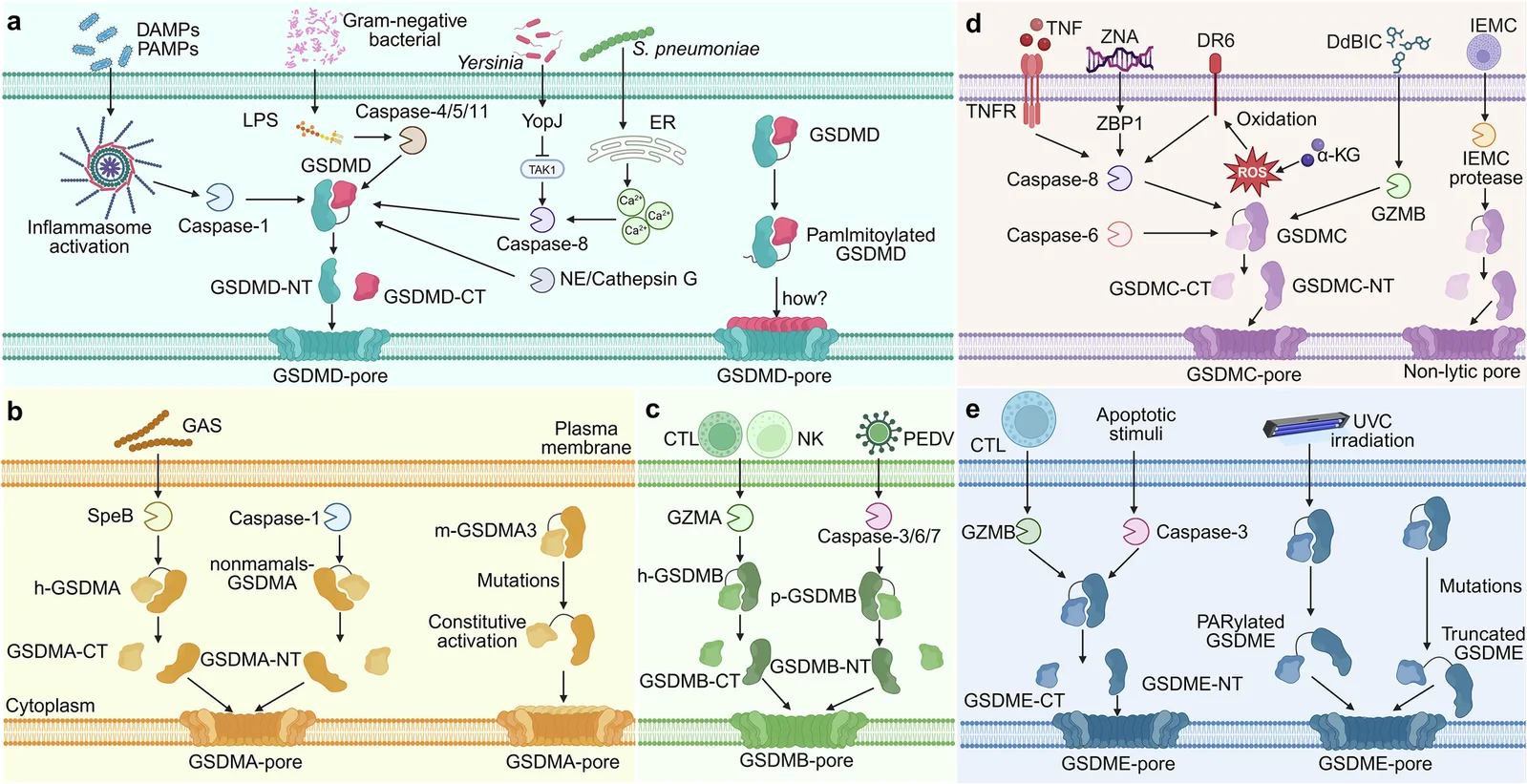
Mechanisms of gasdermin activation. **a** In response to pathogen-associated proteins (PAMPs) or damage-associated molecular patterns (DAMPs), inflammasome assembly activates caspase-1, which cleaves GSDMD to generate a pyroptotic NT fragment. Caspase-4/5/11 is activated by direct binding to intracellular LPS and subsequently cleaves GSDMD. Caspase-8 cleaves GSDMD during *Yersinia* infection (via YopJ-mediated TAK1 inhibition) or *Streptococcus pneumoniae* infection (via ER calcium release). In neutrophils, GSDMD is processed by the serine proteases neutrophil elastase (NE) and cathepsin G. Full-length GSDMD can also be activated by palmitoylation without cleavage, although the specific mechanism remains to be elucidated. **b** During Group A *Streptococcus* (GAS) skin infection, the virulence factor Streptopain (SpeB) cleaves human GSDMA (h-GSDMA) into N-terminal (NT) and C-terminal (CT) fragments, triggering pyroptosis. In non-mammalian species, GSDMA is cleaved by caspase-1 to generate a pore-forming NT fragment. Mutations in mouse *Gsdma3* relieve autoinhibition, leading to constitutive activation of mouse GSDMA3 (m-GSDMA3). **c** Granzyme A (GZMA), a serine protease secreted by cytotoxic T lymphocytes (CTLs) and natural killer (NK) cells, cleaves GSDMB to yield a pore-forming NT fragment. Porcine Epidemic Diarrhea Virus (PEDV) infection triggers caspase-3/6/7-mediated cleavage of porcine GSDMB (p-GSDMB), inducing pyroptosis. **d** Caspase-8 cleaves GSDMC to generate a pore-forming NT fragment in response to tumor necrosis factor (TNF), ZNA, or the metabolite α-ketoglutarate (α-KG). GSDMC is also cleaved by granzyme B (GZMB) following treatment with the compound dodecyl 1H-benzo[d]imidazole-5-carboxylate (DdBIC). Additionally, proteases derived from intraepithelial mast cells (IEMCs) process GSDMC to form specific non-lytic pores. **e** CTL-derived GZMB cleaves and activates GSDME to induce pyroptosis in target cells. Additionally, caspase-3 cleaves GSDME in response to apoptotic signals. In UVC-irradiated tumor cells, GSDME undergoes poly(ADP-ribosyl)ation (PARylation), which relieves autoinhibition and initiates pyroptosis without cleavage. Gain-of-function mutations in GSDME also result in direct activation and pyroptosis. This figure is created in BioRender

Upon activation, these sensors interact with the adaptor protein apoptosis-associated speck-like protein containing a CARD (ASC) and pro-caspase-1 to form the canonical inflammasome complex.^55,60,103,104^ Within the inflammasome complex, pro-caspase-1 undergoes dimerization and autoproteolysis to acquire protease activity.^105,106^ Activated caspase-1 then cleaves GSDMD (Asp276 in mice and Asp275 in humans), liberating the cytotoxic GSDMD-NT to form pores in the membrane (Fig. 1a).^13,14^ Concurrently, caspase-1 cleaves pro-IL-1β and pro-IL-18 into their mature forms, which are released through the GSDMD-NT pores, thereby activating immune and antimicrobial defense.^105,107^ Interestingly, a study suggested that the RFWK motif within the GSDMD-NT β1-β2 loop binds the caspase-1/11 catalytic pocket to inhibit caspase activity and exerts negative feedback control on the inflammasome.^108^

There is also a non-canonical inflammasome pathway that does not involve typical sensor proteins, where murine caspase-11 or human caspase-4/5 directly detect cytosolic gram-negative bacterial lipopolysaccharide (LPS) and are activated (Fig. 1a).^14,109–112^ Mechanistically, the CARD domains of these inflammatory caspases directly bind the lipid A moiety of intracellular LPS.^14,110–112^ It has been reported that this process is regulated by Type I interferon signaling, which upregulates guanylate-binding proteins (GBPs).^113^ Specifically, murine GBP2 and GBP5 rupture pathogen-containing vacuoles to release LPS, while human GBP1 coats cytosolic bacteria to facilitate caspase docking and activation.^113–117^ Like activation in canonical inflammasomes, these caspases undergo dimerization and autoproteolysis to gain catalytic activity.^105^ They subsequently cleave GSDMD at the same residues (Asp276 in mice and Asp275 in humans), liberating cytotoxic GSDMD-NT to induce pyroptosis and potassium efflux. This potassium efflux then acts as a secondary trigger to activate the canonical NLRP3 inflammasome, which is required for the subsequent processing of pro-IL-1β and pro-IL-18 into their mature forms, as non-canonical caspases cannot directly cleave these cytokines.^118–120^

Caspase-4, caspase-5, and caspase-11 also respond to LPS-independent triggers.^121–123^ For instance, the gram-positive bacterium *Streptococcus pyogenes* and its lipoteichoic acid (LTA) can activate caspase-4 and caspase-5 in human monocytes through the MyD88, RIPK1, and caspase-8 axis, rather than through direct ligand binding.^123^ Furthermore, caspase-11 could mediate immune surveillance against eukaryotic pathogens lacking LPS, such as the parasite *Toxoplasma gondii* and the fungus *Aspergillus fumigatus*, although the precise mechanism remains unclear.^121^ Host-derived lipids have also been found to modulate this pathway. Caspase-11 can directly bind and identify oxidized phospholipids to trigger inflammasome activation in dendritic cells.^124^ However, this interaction is context dependent, as oxidized phospholipids and mitochondrial cardiolipin have been observed to inhibit caspase-11 and caspase-4 activation in macrophages by interfering with LPS binding.^125,126^

GSDMD can also be activated through inflammasome-independent pathways. During *Yersinia* infection, the bacterial effector YopJ blocks TAK1 kinase activity, thereby activating caspase-8 for GSDMD cleavage (Fig. 1a).^21,127^ A recent study further indicated that serine/threonine-protein kinase RIO2 (RIOK2) enhances this host defense by facilitating the translocation of the FADD-RIPK1-caspase-8 complex from lysosomes to the endoplasmic reticulum, where it triggers caspase-8-mediated GSDMD cleavage and pyroptosis.^128^ Similarly, calcium, released from the endoplasmic reticulum (ER) during *Streptococcus pneumoniae* infection or cellular states of FADD/XIAP deficiency, can also induce caspase-8 activation (Fig. 1a).^129^ In neutrophils, GSDMD is processed by the serine proteases neutrophil elastase (NE) and cathepsin G (Fig. 1a). NE cleaves GSDMD at Val251 (mouse) or Cys268 (human), and cathepsin G cleaves GSDMD at Leu274.^130–132^ Furthermore, GSDMD can be cleaved at sites that do not cause cell death (mechanisms that will be detailed in the subsequent section). For instance, caspase-3/8 processing releases a p20 fragment essential for bone homeostasis, whereas caspase-3/7 cleavage produces a p13 fragment for food tolerance.^50,51^ In pathogenic contexts, *Leishmania* infection generates a p25 fragment driving NLRP3 activation, whereas allergen exposure induces a p40 fragment that supports IL-33 secretion and type-Ⅱ immunity.^133,134^ A recent study indicated that GSDMD undergoes palmitoylation at Cys191 (human) or Cys192 (mouse), which is crucial for pore formation. Remarkably, this event enables full-length GSDMD to form similar pores even in the absence of cleavage (Fig. 1a). However, the precise molecular mechanism remains to be elucidated.^49^

#### GSDMA

GSDMA, located at 17q21.1, functions as an executioner of pyroptosis, mostly in epithelial barriers, including the skin, esophagus, and bladder.^38,135–138^ The activation mechanism of GSDMA is not well known. It has been reported that human GSDMA lacks susceptibility to caspase cleavage, while non-mammalian homologs in birds and reptiles retain a tetrapeptide sequence that allows for cleavage and activation by caspase-1 (Fig. 1b).^16,139^ However, another study revealed that African swine fever virus (ASFV) infection activates caspase-3 and caspase-4 to cleave porcine GSDMA at Asp75 and Asp241 to inhibit pyroptosis caused by GSDMA-NT, suggesting that GSDMA activation mechanisms may differ significantly across species.^140^ Specifically, Group A *Streptococcus* (GAS) infection secretes the cysteine protease SpeB (streptopain), which directly cleaves human GSDMA at the Gln246 residue (Fig. 1b). This cleavage removes the autoinhibitory GSDMA-CT and liberates the cytotoxic GSDMA-NT (1-246 amino acids), which initiates pyroptosis in skin keratinocytes.^16,17^ In *Gsdma3* mutant mice, mutations causing premature truncation or loss of domain binding resulted in constitutive activation, skin inflammation, and alopecia (Fig. 1b).^16,17,38^

#### GSDMB

GSDMB is encoded on human chromosome 17q21.1 and lacks a murine ortholog.^12^ It is characterized by extensive alternative splicing that generates at least six distinct isoforms.^53,141,142^ Unlike GSDMD, GSDMB functions primarily as a cytotoxin for adaptive immunity. Its canonical activation is triggered by Granzyme A (GZMA), a serine protease delivered into target cells via the immunological synapse formed by cytotoxic T lymphocytes (CTLs) and natural killer (NK) cells (Fig. 1c).^18,25,143^ While it was initially thought that GZMA acts specifically on the longest isoform (Isoform 4), existing evidence indicates that GZMA is capable of cleaving multiple GSDMB isoforms. The variations in the interdomain linker sequence serve to modulate this cleavage efficiency by GZMA.^18,144^ This cleavage releases the cytotoxic N-terminal domain from its C-terminal autoinhibitory domain, facilitating pore formation in the plasma membrane and ensuring the clearance of tumor cells or virus-infected tissues.^25^ A recent study revealed that the interaction between human GZMA and human GSDMB requires GZMA dimerization, which is a unique feature among human granzymes. Disrupting this dimerization abolishes GSDMB binding and the subsequent induction of pyroptosis without affecting GZMA activity against other substrates, indicating a special recognition mechanism for GSDMB. Structural analysis demonstrated that one monomer of the GZMA dimer engages the GSDMB CT via an exosite, while the other positions its catalytic center to cleave the interdomain linker. This configuration ensures precise alignment of the cleavage site (Lys244) with the active site, while shortening the linker by even a single amino acid prevents cleavage. While mouse GZMA adopts a similar dimeric architecture, its exosite engages GSDMB less efficiently. Mutating the mGZMA exosite restores its ability to efficiently cleave and activate GSDMB, offering a highly valuable model for studying the pathophysiological functions of cytotoxic lymphocyte-mediated pyroptotic killing of target cells under various immunological contexts.^145^ However, this immune response can be counteracted by pathogens or modulated by host enzymes in specific situations. The enteric pathogen *Shigella flexneri* utilizes the E3 ubiquitin ligase IpaH7.8 to evade this response. IpaH7.8 binds to GSDMB-NT by recognizing a specific acidic motif (residues Gln15, Asp17, and Asp21) within the α1 helix, targeting the protein for polyubiquitination and subsequent degradation, thereby blocking pyroptosis.^54,146^ Furthermore, GSDMB integrates into broader apoptotic and inflammatory networks with opposing outcomes. While it can enhance non-canonical caspase-4 activity to amplify LPS sensing, it is simultaneously vulnerable to inactivation by apoptotic caspase-3 and caspase-7, which cleave the protein at Asp91 to neutralize its pore-forming ability.^19^ In contrast, a new study indicated that porcine epidemic diarrhea virus (PEDV) infection triggers the cleavage of porcine GSDMB at Asp237 by caspase-3/6/7, thereby inducing pyroptosis (Fig. 1c).^147^ Moreover, one study proposed that caspase-1 could repurpose GSDMB to induce inflammation in the context of asthma; however, this hypothesis remains debated. Independent investigations have not validated caspase-1 as a direct activator of GSDMB.^144,148^

#### GSDMC

GSDMC, mapping on chromosome 8 (8q24.21), is historically known as MLZE.^149^ It is activated through a unique pathway distinct from classical inflammasomes. Although widely expressed in digestive and epithelial tissues, its function is strictly context dependent, acting as a molecular switch between cell death and immune signaling. In the context of cancer, specifically under hypoxia, nuclear PD-L1 cooperates with p-STAT3 to upregulate GSDMC transcription. Subsequently, upon TNF-α stimulation, activated caspase-8 cleaves GSDMC at Asp365 (Fig. 1d).^150^ While the study also found that caspase-6 can cleave GSDMC at the same site, it is not required for TNFα-induced pyroptosis under hypoxia.^150^ Alternatively, the metabolite α-ketoglutarate (α-KG) induces reactive oxygen species (ROS) that oxidize death receptor 6 (DR6) and recruit caspase-8 to cleave GSDMC at Asp240, initiating pyroptosis in gastric and colonic cancer cells (Fig. 1d).^151^ Type III interferons upregulate Z-DNA-binding protein 1 (ZBP1), which recognizes Z-nucleic acids released during intestinal damage, subsequently triggering caspase-8 activation and GSDMC cleavage (Fig. 1d).^152^ A recent study also indicated that GSDMC undergoes cleavage by Granzyme B (GZMB) at Asp276 in response to the chemical compound dodecyl 1H-benzo[d]imidazole-5-carboxylate (DdBIC), which targets the nuclear receptor Nur77 to induce this pyroptotic pathway (Fig. 1d).^153^ Conversely, in intestinal epithelial cells (IECs) during helminth infection, activation by STAT6 signaling or proteases derived from intraepithelial mast cells (IEMCs) triggers GSDMC to form specific non-lytic pores that facilitate the unconventional secretion of the alarmin IL-33, thereby driving type II immunity without causing host cell death (Fig. 1d).^154^

#### GSDME

GSDME, encoded on chromosome 7p15.3 and originally identified as the deafness-associated gene DFNA5, is activated through diverse proteolytic and non-proteolytic mechanisms that extend beyond its initial association with hereditary hearing loss.^23,24,155–162^ While widely expressed in tissues such as the cochlea, brain, and placenta, its activation is mostly caused by specific gain-of-function mutations or protease cleavage. Mutations that cause premature termination of the protein remove the inhibitory CT domain, resulting in constitutively active truncated GSDME that induces cochlear hair cell death and hearing loss.^156–160,162^ In the context of immune signaling and chemotherapy, GSDME is a specific substrate for caspase-3 and GZMB (Fig. 1e).^23,24,155^ Both proteases cleave GSDME at the Asp270 residue, liberating the pore-forming N-terminal fragment to perforate the plasma membrane. GZMB, delivered by cytotoxic lymphocytes, directly activates this pathway to enhance antitumor immunity.^24^ Beyond these classical cleavage events, recent studies have revealed alternative activation modes. Under UVC irradiation, GSDME can undergo poly(ADP-ribosyl)ation (PARylation), allowing it to become active without cleavage (Fig. 1e).^48^ Furthermore, in neutrophils, the serine protease PR3 promotes GSDME processing indirectly by activating caspase-3.^163^ Transcriptional regulation also plays a critical role in licensing this pathway. In atherosclerosis, STAT3 binds to the GSDME promoter to enhance its expression in macrophages, while Sp1 and p53 upregulate GSDME transcription in response to chemotherapy or tumor suppression signals.^164^ However, tumor cells frequently silence GSDME through methylation, which limits the effectiveness of anticancer treatments.^165,166^

#### DFNB59

Pejvakin (DFNB59) stands apart from the rest of the gasdermin family. Encoded on chromosome 2q31.2, it diverges structurally by carrying a truncated C-terminal domain that lacks homology with other family members. It is expressed widely in the brain, lung, and inner ear, but its function is specialized in auditory neurons and hair cells.^167,168^ Unlike typical GSDMs that require cleavage for activation, the mechanism for DFNB59 activation is still not fully understood. Some studies have proposed that DFNB59 is activated directly in response to noise-induced ROS, triggering pexophagy. This selective clearance of damaged peroxisomes is thought to be critical for shielding cochlear hair cells and auditory neurons from oxidative damage.^168–170^ However, the functional activity of DFNB59 remains inconclusive, and its precise role in these processes requires further careful evaluation.^11^ The functional consequence of its truncated C-terminal domain also remains unclear, and it is currently debated whether DFNB59 possesses pore-forming activity similar to other GSDMs.

#### GSDMs in nonmammals

The Gasdermin family extends well beyond mammals, with homologs identified in birds, reptiles, amphibians, corals, fungi, and bacteria, revealing that pyroptosis is an evolutionarily ancient immune strategy. As mentioned above, GSDMA in birds and reptiles can be cleaved by caspase-1 to induce pyroptosis.^139^ A conserved GSDME homolog in corals has been identified and can be cleaved by both coral and human caspase-3 (at Asp241 and Asp257) to form two active NT isoforms, each capable of inducing pyroptosis.^171^ Recent discoveries have identified bacterial homologs of gasdermin (bGSDMs) that execute programmed cell death to defend against phage infection. bGSDMs consist of a conserved pore-forming domain and a short CT domain with an autoinhibitory effect, and they are activated by caspase-like proteases and employ a conserved, lipid-dependent mechanism to form extensive membrane pores.^172,173^ In ancient eukaryotes, *Tricho*GSDM and RCD-1 have been identified as GSDM-like proteins but only have pore-forming domains.^174^ *Tricho*GSDM, identified in the basal metazoan *Trichoplax adhaerens*, is a disulfide-linked autoinhibited dimer and is activated by reduction of the disulfides, indicating a redox-responsive function.^174^ In contrast, RCD-1 from the filamentous fungus *Neurospora crassa* relies on a different mechanism. While individual RCD-1-1 and RCD-1-2 variants bind phospholipids without forming pores, heterorecognition between incompatible alleles triggers cell death.^174,175^ Similarly, in the filamentous fungus *Podospora anserina*, HET-Q1 is identified as a GSDM-like protein that is cleaved by HET-Q2, a subtilisin-like serine protease, during the allorecognition process.^176^

### Pore formation

After activation, GSDM-NTs translocate to the cell membrane, recognize specific lipids and oligomerize to assemble into pores.^177,178^ GSDM-NTs preferentially bind to acidic phospholipids, such as cardiolipin (CL), phosphatidylinositol-4-phosphate (PI4P), phosphatidylinositol-(4,5)-bisphosphate (PI(4,5)P_2_), phosphatidic acid and phosphatidylserine, but do not bind to phosphatidylethanolamine or phosphatidylcholine.^20,31^ Because these acidic lipids are enriched on the inner leaflet of the mammalian plasma membrane, it was traditionally assumed that GSDMs exclusively permeabilize cells from the inside, thereby preventing damage to intact bystander cells and avoiding uncontrolled hyperinflammation. However, recent evidence challenges this paradigm by demonstrating that extracellularly released GSDM fragments can indeed target and permeabilize neighboring cells from the outside. Mechanistically, extracellular vesicles (EVs) released by pyroptotic cells can transplant active GSDM pores directly onto the plasma membranes of naive cells. This EV-driven bystander effect reveals a highly complex, non-cell-autonomous role for GSDMs in disseminating lytic death, amplifying local cytotoxicity, and reshaping the extracellular inflammatory microenvironment.^179^ Moreover, GSDM-NTs also target cardiolipin on mitochondrial membranes, causing mitochondrial damage and enhancing cell death.^180–183^ However, it remains to be demonstrated whether other organelles containing these acidic lipids are similarly targeted.

The monomers of GSDM-NT undergo significant conformational changes and oligomerization to form pores.^1,2,8,46,53^ The GSDM-NT monomer adopts a conformation resembling a left hand.^26,184^ This structure features a β1-β2 loop acting as the ‘wrist’, a globular subdomain functioning as the ‘palm’, and an α1-helix serving as the ‘thumb’. The palm and thumb domains assemble to form the crown of the GSDM pore, while the ‘fingers’, composed of antiparallel β-hairpins with four long β-strands (15–22 amino acids), insert into the lipid membrane.^26,53,54,184^ Upon membrane insertion, the conformation of the GSDM subunit in the pore structure changes. The “fingers” (transmembrane β-hairpins) extend to penetrate the lipid bilayer, while the globular subdomain remains cytosolic.^26,53,54,184^ Structural analysis reveals a flexible angle between these two domains that varies across homologs. This angle is widest in GSDMD, followed by GSDMA3 and GSDMB, and is narrowest in bGSDM.^26,53,173^ The β1-β2 loop and adjacent basic patches on the GSDM subunits facilitate membrane lipid binding. Multiple protomers assemble to form a large β-barrel pore, which is mediated by extensive inter-strand hydrogen bonding within the membrane, as well as by specific hydrophobic and hydrogen-bonding interactions between the cytosolic globular domains.^26,53,54,184^ This oligomerization pattern is conserved across all GSDMs with pore structures, including GSDMD, GSDMA3, and GSDMB. It represents the typical mode of assembly for immobile, membrane-inserted structures, which manifest variously as arcs, slits, or complete rings. Researchers have also observed that GSDMD-NT and GSDMA3-NT can form smaller arc-shaped and slit-shaped pores, which are composed of as few as two monomers.^185,186^ While these minimal assemblies allow the passage of water and ions, they can grow and fuse into larger arc-shaped pores capable of transporting small proteins across the plasma membrane. The pores may be compressed into narrower slit-shaped channels by lipid bilayer tension or, conversely, continue to expand and oligomerize into complete ring-shaped channels.^185–187^ In contrast to the insertion-first model, other studies on GSDMD and GSDMA3 have identified mobile, ring-shaped oligomers that assemble on the membrane surface prior to insertion.^188–192^ However, it has not been proven that these non-inserted rings could penetrate the bilayer to become functional pores. Taken together, these findings suggest that GSDM may either directly insert to create small arcs or slits, which then fuse with other subunits to grow into large pores, or pre-assemble into a complete ring on the intracellular membrane surface.

Cryo-EM studies have revealed that human GSDMD-NT pores are composed of 31–34 subunits with an inner diameter of 20–21.5 nm. In comparison, GSDMA3 pores comprise 26–28 subunits (18 nm inner diameter), and GSDMB pores consist of 26–30 subunits.^26,53,54,184^ Recent high-resolution cryo-EM analyses have expanded this structural landscape by elucidating the GSDME pore, which assembles from 27 to 28 subunits. Notably, GSDME forms an extended transmembrane β-barrel, which is the longest reported to date within the gasdermin family. Each of its β-strands comprises approximately 14 residues (compared to 11–12 in other homologs), enabling the GSDME pore to span a thicker lipid bilayer of ~40 Å.^193^ The pore diameter is sufficient to pass larger molecules, such as IL-1 family cytokines, galectin-1/3, ATP, and the cold-inducible RNA binding protein (CIRP).^134,177,194–196^ It is interesting that GSDMD pores preferentially release mature IL-1 over pro-IL-1 despite their similar molecular dimensions, both markedly smaller than the pore. Research indicates that this regulation is governed by the electrostatic environment of the pore.^197^ Moreover, bGSDM pores tend to be larger and more variable in size. For instance, *Bacteroidetes* GSDM pores consist of 30–31 protomers with an inner diameter of approximately 18 nm, and *Vitiosangium* pores are significantly wider, containing 48–54 protomers with an inner diameter of approximately 28 nm.^173^

### Cell rupture

Recent evidence has shown that Ninjurin-1 (NINJ1) is responsible for plasma membrane rupture.^198,199^ In healthy cells, NINJ1 is located on the plasma membrane as auto-inhibited dimers.^199^ Upon receiving cell death signals, such as calcium influx or mechanical tension, NINJ1 polymerizes into extensive oligomeric filaments that assemble into rings ranging in size from nanometers to micrometers. These filaments possess a hydrophobic concave side facing the membrane and a hydrophilic convex side facing the extracellular space, disrupting the lipid layer of the plasma membrane.^198–207^ A recent biophysical model proposes that NINJ1 oligomerizes into double-filament structures, which are subsequently pulled apart by swelling-induced membrane tension to create large rupture lesions. This mechanical dependency explains why its homolog, NINJ2, fails to induce membrane excision. Although NINJ2 also oligomerizes, its highly stable dimer lacks the unstructured N-terminal region necessary to respond to such tension.^208^ Cells with NINJ1 deficiency do not rupture but consistently exhibit a balloon-like bubble morphology.^198^ Consequently, NINJ1 is essential for the release of large DAMPs, such as HMGB1, LDH, and cytosolic proteins, that cannot pass GSDM pores.^198^ By releasing these DAMPs, NINJ1 spreads inflammation and recruits immune cells such as neutrophils and macrophages to the site of injury.^199,202–205^ A recent study revealed that pyroptotic cell swelling occurs in two distinct phases. In the first phase, GSDMD initiates the process by forming oligomeric arc- or slit-like pores. These pores are permeable to ions and small molecules (10–70 kDa), driving initial cell swelling that stabilizes into a transient volume plateau. In the second phase, NINJ1 is recruited to mediate rapid pore expansion, enabling the influx of larger molecules and culminating in plasma membrane rupture.^209^ Notably, NINJ1 is required not only for terminal rupture but also for the maintenance of the GSDMD-induced plateau, implying functional coupling via membrane tension rather than direct protein‒protein interactions. Furthermore, Ca²⁺ influx via GSDMD pores accelerates NINJ1 oligomerization, effectively overriding ESCRT-mediated membrane repair to ensure cell death.^210,211^ NINJ1-mediated rupture is critical for clearing intracellular bacterial infections. For instance, mice lacking NINJ1 are highly susceptible to infection by *Citrobacter rodentium*.^198^ The rupture also exposes cytoskeletal elements such as F-actin, which can be recognized by dendritic cell receptors (DNGR-1) to initiate antigen cross-presentation and adaptive immune responses.^212^ As NINJ1 drives the inflammatory phase of cell death, it is a promising therapeutic target for diseases driven by excessive necrosis or inflammation. NINJ1 inhibition has demonstrated protective effects in models of liver ischemia‒reperfusion injury, fulminant hepatitis, and sepsis.^206,213^ The precise molecular mechanism and physiological implications of NINJ1-mediated plasma membrane disruption remain to be fully elucidated.

### Membrane repair

It is worth noting that not all cells with GSDM pores will eventually lyse. Phagocytes can achieve a state of hyperactivation, which is defined by their ability to secrete IL-1 while retaining viability.^177,214^ While the precise mechanisms sustaining this state remain under investigation, calcium influx through GSDMD pores has been identified as a critical survival signal. This influx recruits the endosomal sorting complexes required for transport (ESCRT) machinery to repair the damaged plasma membrane.^215^ Consequently, inhibiting ESCRT-III strongly enhances pyroptosis and IL-1β release during inflammasome activation, avoiding overt inflammation.^215^ Capsaicin has been found to activate ESCRT-III-dependent membrane repair via TRPV1-mediated calcium influx, thereby mitigating acute alcohol-induced pyroptosis.^216^ Another study demonstrated that GSDMD pores are dynamic structures and that their opening and closing kinetics are modulated by pore-mediated calcium influx and phosphoinositide metabolism.^217^ Researchers have also found that caspase-7 preserves cell integrity by cleaving and activating acid sphingomyelinase (ASM), triggering ceramide generation to enhance membrane repair.^218^ Recently, a new study showed that the duration of pore permeability is a decisive factor in cell fate. While a limited duration (<2 h) of membrane GSDMD pores is not detrimental, a longer duration (21 h) of pore formation leads to extensive membrane damage and cell death. In this context, ALIX has been identified as a key regulator of GSDMD-NT clearance and the restoration of membrane integrity.^219^ Apart from mitigating pyroptosis, ESCRT-III also sustains cell survival during MLKL-mediated necrosis. This repair process extends cell survival, preserves membrane integrity, and promotes the antigenic cross-priming of CD8^+^ T cells.^220^ Phospho-MLKL is eliminated from the plasma membrane through flotillin-dependent endocytosis or ALIX-syntenin-1-mediated exocytosis, effectively inhibiting necroptosis-induced membrane rupture.^221^ These repair processes are critical for regulating inflammation and antitumor immunity, although their specific mechanisms require further investigation.

### Post-translational modifications of gasdermins

Post-translational modifications (PTMs) of GSDMs have been extensively explored, revealing that these modifications can affect both pyroptotic and non-pyroptotic functions (Table 2).

**Table 2:** PTMs modulating the activities of the GSDM family

GSDM	Modification type	Site (Species)	Enzyme/Modifier	Function	Refs
GSDMA	Phosphorylation	Thr8 (human)	PLK1	Inhibits pore formation	^36^
	Phosphorylation	Ser353 (human)	ULK1	Activates GSDMA without it being cut, causing pyroptosis	^222^
	Palmitoylation	Not confirmed	Not confirmed	Not confirmed	^49^
GSDMB	Ubiquitination	Not confirmed	IpaH7.8 (K48-linked)	Targets GSDMB for degradation	^146^
	Deubiquitination	Not mentioned	USP24	Stabilizes the protein	^223^
	Palmitoylation	Not confirmed	Not confirmed	Not confirmed	^49^
GSDMC	Palmitoylation	Not confirmed	Not confirmed	Not confirmed	^49^
GSDMD	Palmitoylation	Cys191 (human)/Cys192 (mouse)	ZDHHC5/7/9	Promotes GSDMD activation, movement to the cell membrane, and pore formation	^49,224–226^
	Depalmitoylation	Cys191 (human)/Cys192 (mouse)	APT2	Enables GSDMD-NT oligomerization	^225^
	Oxidation	Cys191 (human)/Cys192 (mouse)	ROS	Potentiates the oligomerization of GSDMD-NT and enhances pore formation	^228^
	Oxidation	Cys38/56/268/467 (human)/Cys39/57/265/487 (mouse)	ROS	Promotes the cleavage of GSDMD	^229^
	Phosphorylation	Ser46 (human)/Ser47 (mouse)	AMPK	Inhibits GSDMD from moving to the membrane and forming pores, blocking pyroptosis	^230^
	Phosphorylation	Thr213 (human)	PP1	Inhibits active GSDMD from assembling into pores	^231^
	Ubiquitination	Lys55, Lys62, Lys203 (human)	IpaH7.8 (K48/K63-linked)	Targets GSDMD for degradation, blocking pyroptosis and helping bacteria survive	^232^
	Ubiquitination	Lys203/204 (human)	SYVN1 (K27-linked)	Promotes pyroptosis	^233^
	Ubiquitination	Lys168 (human/mouse)	MIB2 (K33-linked, recruited by USP18)	Targets GSDMD for degradation, inhibiting pyroptosis	^234^
	Ubiquitination	Lys51, Lys168 (human/mouse)	RING1 (K48-linked)	Targets GSDMD for degradation, inhibiting pyroptosis	^235^
	Ubiquitination	Lys51, Lys203, Lys 204 (human)	NEDD4L	Targets GSDMD for degradation, inhibiting pyroptosis	^236^
	Ubiquitination	Lys51 (human)	TRIM21	Targets GSDMD for degradation, inhibiting pyroptosis	^237^
	Deacetylation	Lys248 (human)	HDAC4	Promotes ubiquitination to enhance pyroptosis	^241^
	SUMOylation	Lys177 (human)	/	Stabilizes GSDMD and promotes pyroptosis	^242^
	O-GlcNAcylation	Ser338 (human)	OGT	Reduces pyroptosis by blocking the interaction needed for GSDMD cleavage	^243^
	Succination	Cys191 (human)/Cys192 (mouse)	Fumarate	Blocks the cleavage and pore formation of GSDMD, inhibiting pyroptosis	^244^
	Itaconation	Cys77 (mouse)	Itaconate	Prevents GSDMD from being cleaved by caspase-1	^245^
GSDME	Palmitoylation	Cys180 (Primary), Cys45 (Auxiliary) (human)	/	Enhances GSDME pore-forming efficiency	^193^
	Palmitoylation	Cys407/Cys408 (human/mouse)	ZDHHC2/7/11/15	Helps activate GSDME to form pores during chemotherapy	^246^
	Phosphorylation	Thr6 (human)	AMPK	Blocks both the cutting and pore formation of GSDME	^247^
	Ubiquitination	Lys39, Lys40, Lys120, Lys440 (human)	NEDD4L	Targets GSDME for degradation, inhibiting pyroptosis	^236^
	Ubiquitination	Not mentioned	CDC20 (K48-linked)	Targets GSDME for degradation	^248^
	Deubiquitination	Not mentioned	OTUD4	Stabilizes the protein	^249^
	Deubiquitination	Lys 120, Lys 189	USP48 (K48-linked)	Stabilizes the protein	^250^
	Succination	Cys45 (mouse)	Fumarate	Blocks GSDME cleavage and pore formation	^244^
	PARylation	Not mentioned	PARP5	Relieves autoinhibition, enabling cleavage-independent activation by lipid ROS	^48^

#### GSDMA

GSDMA is phosphorylated at two distinct sites, Thr8 and Ser353, by two different kinases with opposing effects. While the protein kinase ULK1 phosphorylates Ser353 to disrupt GSDMA’s auto-inhibitory structure, PLK1 phosphorylates Thr8 to inhibit its pore-forming activity.^36,222^

#### GSDMB

GSDMB is ubiquitinated by the *Shigella* protein IpaH7.8, which targets it for proteasomal degradation, allowing the bacteria to evade NK cell-mediated killing.^146^ Another study showed that GSDMB is deubiquitinated by USP24, which stabilizes the protein and prevents its degradation. This stabilization allows GSDMB to interact with and activate STAT3 signaling. The activation of the USP24/GSDMB/STAT3 axis ultimately promotes the growth and invasion of bladder cancer cells, suggesting a potential new target for therapeutic intervention.^223^

#### GSDMD

S-palmitoylation, the reversible attachment of the 16-carbon fatty acid palmitate to cysteine residues, is a critical PTM for GSDMD function. A study showed that GSDMD is palmitoylated at Cys191 by the enzymes ZDHHC5 and ZDHHC9 and that palmitoylation is required for pore formation. This research also makes the striking discovery that this palmitoylation enables even the uncleavable, full-length GSDMD with the D275A mutation to form pores similar to those of the cleaved GSDMD-NT fragment. Furthermore, the study indicates that other human GSDMs are also palmitoylated at their N-terminal domains, with GSDME uniquely being modified at its C-terminal domain as well.^49^ Studies consistently support that GSDMD palmitoylation at Cys191 (human)/Cys192 (mouse) is crucial for promoting pyroptosis by facilitating protein membrane localization. While some research identifies ZDHHC5 and ZDHHC9 as the primary enzymes responsible for this modification, others point to ZDHHC7.^49,224–226^ While palmitoylation of GSDMD is known to promote pyroptosis, the removal of this modification is also crucial. Specifically, APT2-mediated depalmitoylation plays a critical role in the oligomerization of the GSDMD-NT fragment. This suggests a complex regulatory cycle where both the addition and removal of palmitate are essential for controlling the pyroptotic process.^225^ The cellular redox state is a key determinant of GSDMD activity. ROS, which are often generated during inflammatory responses, can directly promote GSDMD function.^227^ Oxidation of GSDMD, particularly at the same cysteine residue targeted by palmitoylation, has been shown to potentiate the oligomerization of GSDMD-NT and enhance pore formation.^228^ Notably, ROS have been shown to promote GSDMD Cys191 (human)/Cys192 (mouse) palmitoylation, which is critical for GSDMD activation.^49^ However, ROS-mediated oxidation of Cys191/192 does not affect its membrane translocation, suggesting that the pro-pyroptotic effects of ROS are linked to palmitoylation but are not solely dependent on this modification.^228^ Another study showed that ROS-mediated oxidation of murine GSDMD at Cys39, Cys57, Cys265, and Cys487 promotes GSDMD cleavage by caspase-1 rather than influencing oligomerization.^229^

AMP-activated protein kinase (AMPK) can directly phosphorylate the NT domain of GSDMD at Ser46 (mouse), hindering the oligomerization of GSDMD-NT, effectively abolishing its pore-forming ability and blocking pyroptosis.^230^ The dephosphorylation of GSDMD has also been shown to promote pyroptosis. Research highlights that protein phosphatase 1 (PP1), particularly its catalytic subunits PP1α and PP1γ, dephosphorylates GSDMD at Thr213, a key event that facilitates the execution of pyroptosis.^231^

Multiple E3 ligases ubiquitinate GSDMD. For example, the *Shigella* protein IpaH7.8, which ubiquitinates GSDMB, also binds both human and murine GSDMD. However, it only ubiquitinates and degrades human GSDMD. This selective targeting suggests a potential evolutionary advantage for mice, enabling them to utilize GSDMD-induced pyroptosis as a robust defense mechanism to combat *Shigella* infection.^232^ In addition, Synoviolin 1 (SYVN1) has been reported to ubiquitinate human GSDMD at Lys203/204 with K27-linked chains, which promotes pyroptosis.^233^ Conversely, MIB E3 ubiquitin protein ligase 2 (MIB2) is recruited to GSDMD by ubiquitin-specific peptidase 18 (USP18). This is a particularly interesting interaction because USP18 is a deubiquitinase, yet it facilitates this process through a mechanism that is independent of its own enzymatic activity. Once recruited, MIB2 promotes K33-linked ubiquitination of GSDMD at the Lys168 residue. This specific ubiquitination event acts as a signal for the autophagic degradation of GSDMD, a process that ultimately leads to the inhibition of pyroptosis.^234^ A recent study also identified that human RING1 promotes K48-linked ubiquitination of GSDMD at sites Lys51 and Lys168. This leads to proteasomal degradation and thereby inhibits pyroptosis.^235^ NEDD4L inhibits pyroptosis by promoting the ubiquitination and subsequent degradation of GSDMD at sites Lys51, Lys203, and Lys204, thereby preventing its intracellular accumulation.^236^ Scutellarin has been found to facilitate the K33-linked ubiquitination of GSDMD at residue Lys51, a reaction catalyzed by the E3 ligase tripartite motif-containing 21 (TRIM21). This specific ubiquitination serves as a recognition signal for SQSTM1/p62, which subsequently targets GSDMD for autophagic degradation.^237^ Although the ubiquitination of GSDMD has been studied, the specific deubiquitinase responsible for GSDMD has not yet been identified. Acetylation is a post-translational modification with reported connections to ubiquitination.^238–240^ A study indicated that GSDMD is acetylated at Lys248, which enhances pyroptosis. While the specific acetyltransferases responsible for this modification have not yet been identified, histone deacetylase 4 (HDAC4) has been pinpointed as a deacetylase that mediates the removal of this acetyl group. GSDMD acetylation promotes its ubiquitination, which in turn facilitates pyroptosis.^241^ GSDMD undergoes SUMOylation at residue Lys177, a modification that stabilizes the protein and promotes pyroptosis. Conversely, SUMO-specific protease 3 (SENP3) counteracts this process by removing SUMO conjugates from GSDMD, thereby inhibiting pyroptosis.^242^

A recent study also demonstrated a novel post-translational modification of GSDMD and elucidated that O-GlcNAcylation of GSDMD at S338 inhibits its cleavage by blocking its interaction with caspase-4/5/11. This modification attenuates LPS-induced pyroptosis and juxtacrine signaling in endothelial cells.^243^

The metabolic state of a cell directly influences GSDMD activity through covalent modification by metabolic intermediates. For example, fumarate, an intermediate of the Krebs cycle that accumulates in activated macrophages, acts as an electrophile. It modifies cysteine residues on proteins via a process called succinylation (or succination), and GSDMD is a direct target. In human GSDMD, this modification occurs at multiple cysteine residues (Cys56, Cys191, Cys268, Cys309, and Cys467). The succination of Cys191 is particularly critical, as it inhibits the interaction between GSDMD and its activating caspases. This prevents GSDMD cleavage and effectively blocks pyroptosis, even when caspase activation is intact. This mechanism helps explain the anti-inflammatory effects of dimethyl fumarate (DMF), a drug used to treat multiple sclerosis. In addition to its effect on GSDMD, fumarate can also decrease the cleavage of GSDME, likely through the succination of its reactive cysteines. This suggests that fumarate-mediated succinylation is a broad regulatory mechanism for gasdermin family proteins.^244^ Itaconate is another immune-regulatory metabolite produced in large quantities by activated myeloid cells. Similar to fumarate, itaconate is an electrophile that can covalently modify proteins. Itaconate is known to inhibit NLRP3 inflammasome activation and pyroptosis. Evidence suggests that it can also directly modify GSDMD, potentially at Cys77, which would prevent its full processing and activation.^245^ Both succinylation and itaconation represent powerful negative feedback loops. In these mechanisms, the metabolic changes that occur in inflammatory cells generate metabolites that directly restrain the pyroptotic machinery. This highlights a crucial link between cellular metabolism and the regulation of inflammatory cell death.

#### GSDME

GSDME is also regulated by its own distinct mechanisms. A study revealed that palmitoylation of GSDME-CT at residues Cys407 and Cys408 promotes its dissociation from GSDME-NT. This specific modification, catalyzed by ZDHHC2, 7, 11, and 15, releases GSDME-NT to form pores, inducing pyroptosis in response to chemotherapy.^246^ Recent analyses have revealed that activated GSDME-NT undergoes S-palmitoylation primarily at Cys180. Analogous to GSDMD C191, Cys180 is located within the transmembrane region, indicating a conserved mechanism to reinforce membrane engagement. Moreover, Cys45, which is situated at the lipid-binding tip exposed upon cleavage, plays an auxiliary role. Cys45 palmitoylation enhances membrane attachment in specific contexts but is not strictly required for pore formation, as its individual mutation minimally impairs pyroptosis.^193^ A study indicated that mannose metabolism, through the hexosamine biosynthetic pathway, increases the levels of the metabolite N-acetylglucosamine-6-phosphate (GlcNAc-6P). This metabolite facilitates the phosphorylation of AMPK by LKB1, leading to its activation. Activated AMPK then phosphorylates GSDME at Thr6, which blocks GSDME cleavage by caspase-3 and represses pyroptosis. The study also found that administering mannose suppressed pyroptosis in the small intestine and kidneys, alleviating cisplatin- or oxaliplatin-induced tissue toxicity without compromising their antitumor effects.^247^ The ubiquitination and deubiquitination of GSDME are critical for its regulation. NEDD4L facilitates the ubiquitination of GSDME, driving its degradation and subsequently inhibiting pyroptosis.^236^ CDC20 is a negative regulator that tags GSDME for proteasome degradation. When CDC20 levels are low, GSDME accumulates, causing cells to switch from apoptosis to pyroptosis. This enhances antitumor immunity in mice by increasing CD8^+^ T cells and decreasing immunosuppressive myeloid cells.^248^ OTUD4 and USP48 are deubiquitinases that stabilize GSDME. OTUD4 enhances pyroptosis and radiosensitizes NPC cells by removing ubiquitin from GSDME.^249^ Similarly, USP48 promotes pyroptosis by binding to GSDME and removing K48-linked ubiquitination at positions Lys120 and Lys189, preventing its degradation.^250^

A recent study described a novel mechanism for GSDME activation independent of proteolytic cleavage. DNA damage from UV-C irradiation activates nuclear PARP1, which releases PAR polymers into the cytoplasm. These polymers activate PARP5, which in turn adds a PARylation tag to full-length GSDME. This modification primes GSDME by relieving its autoinhibitory state. Simultaneously, UV-C irradiation increases lipid ROS. Primed GSDME senses this oxidative stress, leading to its oligomerization and subsequent targeting to the plasma membrane, where it forms pores and induces pyroptosis without the need for cleavage.^48^

However, the outcomes of PTMs across GSDMs also depend on domain-specific structural features. Although GSDMs share a conserved architecture, structural differences exist in surface charge distribution, interdomain linker composition, and NT-CT interaction geometry, providing a mechanistic basis for differential PTM sensitivity among GSDMs. PTMs occurring within the NT domain, such as phosphorylation, palmitoylation, oxidation, or metabolic cysteine modification, are likely to influence membrane binding, oligomerization, and pore assembly. Importantly, the extent to which such PTMs promote or inhibit pore formation likely varies among GSDMs due to differences in electrostatic surface properties and oligomerization interfaces within the NT domain. In contrast, PTMs localized to the interdomain linker or its vicinity predominantly regulate protease accessibility and cleavage efficiency. Variability in linker length, flexibility, and exposure among GSDMs may explain why identical or analogous modifications exert distinct effects on activation or cleavage. In this context, PTMs such as O-GlcNAcylation, succination, or itaconation can effectively block the interaction between GSDMs and caspases through steric or chemical interference. Specific PTMs, such as palmitoylation, can directly activate GSDMD without cleavage. Taken together, PTMs act as context-dependent regulatory signals that depend on the domain architecture and structural variation across GSDMs. Incorporating structural considerations into the analysis of GSDM PTMs provides a comprehensive framework to explain how closely related family members execute diverse pyroptotic and non-pyroptotic functions under different physiological and pathological conditions.

## Gasdermins in other programmed cell death pathways

The function of GSDMs extends beyond their established role in executing pyroptosis. Recent studies have indicated that GSDMs exhibit significant functional plasticity, placing them at the intersection of multiple cell death pathways. Rather than acting as simple on/off switches, GSDMs function as signaling hubs that process diverse cellular signals to determine cell fate, ranging from survival to death.^251^

### Gasdermins in apoptosis

**Fig. 2:**
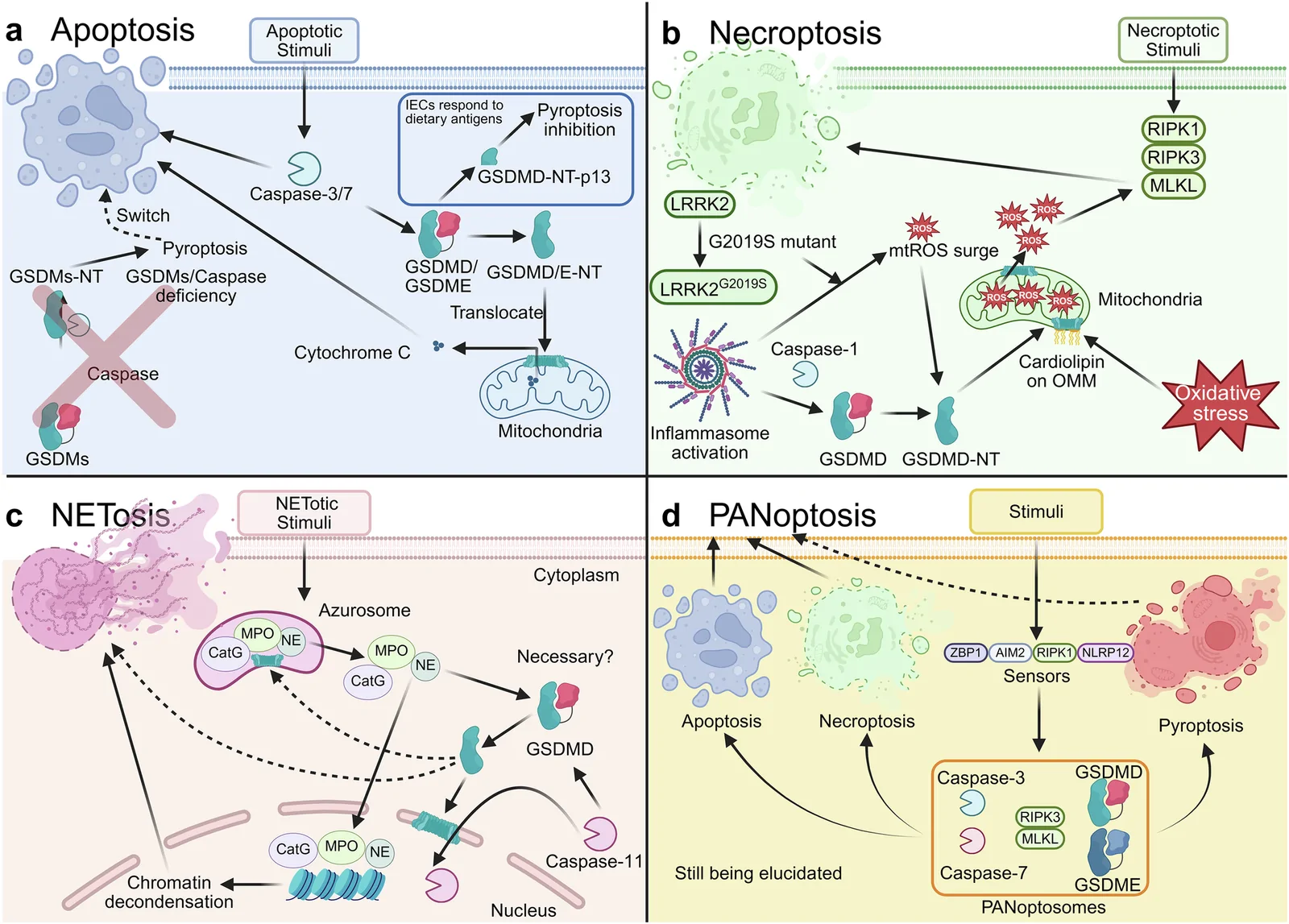
GSDMs in apoptosis, necroptosis, NETosis, and PANoptosis. **a** Upon apoptotic stimuli, caspase-3/7 are activated to induce apoptosis. Caspase-3/7 also cleave GSDMD/GSDME, forming pores in the mitochondrial membrane, which releases cytochrome C to further activate caspase-3, thereby accelerating apoptosis. Human caspase-3/7 cleaves GSDMD at Asp87 to produce GSDMD-NT-p13, which is unable to form pores in the cell membrane, effectively inhibiting pyroptosis. When GSDMs or caspases are absent or inactivated, the cell death pathway can shift from pyroptosis to apoptosis. **b** In macrophages with the Lrrk2^G2019S^ mutation, elevated mitochondrial reactive oxygen species (mtROS) redirect GSDMD to the mitochondria to form pores and release more mtROS, while severe oxidative stress exposes cardiolipin on the outer mitochondrial membrane (OMM) to sequester the GSDMD-NT fragment, with both processes promoting a switch from pyroptosis to necroptosis. **c** While the exact role of GSDMD in NETosis is debated, it is proposed that a cascade of events leads to the formation of NETs. Neutrophil elastase (NE) first cleaves GSDMD to create the active GSDMD-NT fragment, which forms pores to release more NE and ultimately creates pores in the nuclear envelope and plasma membrane to facilitate chromatin decondensation, expansion, and the final explosive release of NETs. **d** PANoptosis is a regulated cell death pathway, distinct from apoptosis, pyroptosis, and necroptosis, that is controlled by the assembly of a single complex called the PANoptosome. This complex activates caspase-3/7, GSDMD/GSDME, and RIPK3/MLKL to initiate each of these death programs, demonstrating their interconnected nature where the suppression of one pathway can be compensated for by another. This figure is created in BioRender

Apoptosis is a form of programmed cell death that is primarily mediated by apoptotic caspases such as caspase-3, −6, −7, −8, and −9. This process is initiated either through extrinsic pathways (like death receptor ligation) or intrinsic pathways (like DNA damage).^252,253^ In response to apoptotic signals, cells initiate a specific cascade. This process leads to the activation of initiator caspases, which then trigger effector caspases to cleave cellular substrates, resulting in controlled cell death. The relationship between the GSDM family and apoptosis is a two-way interaction. Depending on the cellular context and the specific signal, GSDMs can either amplify the apoptotic pathway or be neutralized by it (Fig. 2a).^254^

Upon cleavage of GSDME by caspase-3, a portion of the resulting GSDME-NT fragment translocates to the mitochondria. This translocation leads to the release of cytochrome C and subsequent activation of caspase-3, thereby enhancing apoptosis (Fig. 2a). This mitochondrial targeting and permeabilization are not unique to GSDME, as the inflammasome-generated GSDMD-NT fragment has also been demonstrated to exert similar effects on mitochondrial membranes.^23,36^ Interestingly, in colorectal cancer, nuclear GSDMD actively inhibits cancer cell growth and promotes apoptosis. This effect is mediated by its interaction with PARP-1, a protein critical for maintaining genomic integrity through DNA repair. Nuclear GSDMD compromises PARP-1 function, thereby leading to the accumulation of DNA damage and subsequent induction of cell death.^255^ In intestinal epithelial cells, caspase-3 and caspase-7 cleave GSDMD at Asp87 (human), generating a specific GSDMD-NT-p13 fragment in response to dietary antigens. This p13 fragment translocates to the nucleus to induce MHCII transcription, a process critical for the induction of type 1 regulatory T (Tr1) cells and the maintenance of food tolerance. This fragment lacks the ability to form pores, thereby uncoupling this nuclear function from the protein’s pyroptotic potential.^50^ GSDMB-NT (1–91 aa) has a novel cell-autonomous function. GSDMB can bind to caspase-4, promoting noncanonical pyroptosis. However, caspase-7 cleaves GSDMB at the Asp91 site, which blocks this function. Mechanistically, the resulting GSDMB CT (92–417 aa) binds to GSDMB-NT (1–91 aa), inhibiting its activity.^19^

The expression and activation of GSDMs appear to be critical switches determining whether a cell undergoes pyroptosis or apoptosis. When GSDMs are present and rapidly activated, the cell death pathway can shift from apoptosis to pyroptosis. GSDMC and GSDME have been identified as substrates of apoptotic caspase-8 and caspase-3, respectively, and their cleavage induces pyroptosis at a significantly accelerated rate compared to the typical timeline for apoptosis.^150,155^ Beyond apoptotic caspases, GZMA and GZMB, proteases secreted by cytotoxic T lymphocytes (CTLs) that typically initiate apoptosis, have also been found to directly process and activate GSDMB and GSDME and trigger pyroptosis in cells expressing these specific GSDMs.^25,256^ The absence of key pyroptosis mediators can also shift the pathway toward apoptosis. In GSDMD-deficient macrophages, activation of caspase-1 has been observed to induce apoptosis, a process accompanied by caspase-3 activation and involving the Bid-caspase-9-caspase-3 axis (Fig. 2a).^257^

### Gasdermins in necroptosis

Necroptosis is a programmed form of necrotic cell death driven by a specific kinase cascade involving Receptor-Interacting Protein Kinase 1 (RIPK1), RIPK3, and their downstream substrate, the pseudokinase Mixed Lineage Kinase Domain-Like (MLKL).^258–261^ While the role of GSDMD in necroptosis has been investigated, direct information regarding the involvement of other GSDMs in this specific cell death pathway remains less characterized. The choice between GSDMD-mediated pyroptosis and GSDMD-facilitated necroptosis is a dynamic process driven by a delicate balance of intracellular signals, including ROS levels and mitochondrial integrity, which together influence the localization of GSDMD-NT (Fig. 2b).

The localization of GSDMD-NT is subject to molecular competition, influenced by its binding affinities for lipids in distinct cellular compartments. While phosphatidylinositol phosphates (PIPs) on the inner leaflet of the plasma membrane are the canonical targets for pyroptosis, cardiolipin on mitochondrial membranes serves as an alternative. The condition of the cell determines the outcome of this binding competition. In healthy cells, the plasma membrane is the primary target. However, under severe oxidative stress, mitochondrial damage can lead to the externalization of cardiolipin on the outer mitochondrial membrane (OMM), creating a high-affinity docking site. This creates a strong binding site that traps GSDMD-NT away from the plasma membrane, redirecting it to start the necroptosis pathway (Fig. 2b). In macrophages carrying the Lrrk2^G2019S^ mutation, inflammasome activation can lead to elevated mtROS. This surge in mtROS then directs GSDMD to mitochondrial membranes, initiating GSDMD pore formation and subsequent mtROS release, which ultimately promotes a switch to RIPK1/RIPK3/MLKL-dependent necroptosis (Fig. 2b).^262^ It has also been found that disrupting cardiolipin synthesis or OMM externalization has no effect on apoptosis but significantly attenuates necroptosis.^180^ Additionally, MLKL-driven plasma membrane damage can indirectly activate GSDMD by inducing potassium efflux, which subsequently activates the NLRP3 inflammasome.^263,264^

### Gasdermins in NETosis

Neutrophil extracellular trap (NET) formation, or NETosis, is a unique form of cell death specific to neutrophils, characterized by the extrusion of decondensed chromatin decorated with granular proteins to ensnare and kill pathogens.^265^ The involvement of GSDMs, particularly GSDMD, in the process of NETosis has been controversial.

Early studies proposed a direct role for GSDMD in facilitating NETosis by forming pores at various subcellular locations. During the activation of NETosis, azurophilic granules fuse with phagosomes or release their contents into the cytosol. These contents include potent neutrophil serine proteases (NSPs), most notably neutrophil elastase (NE) and Cathepsin G (CatG) (Fig. 2c).^266–269^ Studies have indicated that GSDMD and caspase-11 are crucial for both the early and late stages of NETosis.^270^ Beyond their role in the final rupture of the neutrophil plasma membrane during NET extrusion, these proteins are vital for initial events such as nuclear delobulation and DNA expansion.^270^ Studies have also shown that NE, in particular, can directly cleave GSDMD to generate the active GSDMD-NT fragment, bypassing the need for canonical inflammasome activation.^130,271^ It is proposed that GSDMD-NT first forms pores on the membranes of granules, facilitating a feed-forward loop by promoting the further release of NE into the cytosol.^271^ NE can then translocate into the nucleus, where it proteolytically cleaves histones, a critical step for chromatin decondensation (Fig. 2c). Then, GSDMD-NT forms pores in both the nuclear envelope and the plasma membrane. Pores in the nuclear membrane are believed to be necessary for the expansion of decondensed chromatin, while plasma membrane pores are essential for the final, explosive release of NETs into the extracellular space, leading to neutrophil lysis.^271,272^ A recent study also supports this, demonstrating that activation of the NLRP3/GSDMD axis significantly enhances NET release from neutrophils during traumatic brain injury.^273^

However, a significant and growing body of research argues that GSDMD is largely dispensable for NETosis.^274,275^ GSDMD-deficient neutrophils can still form NETs just as effectively as normal neutrophils when stimulated with phorbol 12-myristate 13-acetate (PMA), a common drug used to trigger NETosis, and PMA stimulation does not lead to cleavage of GSDMD into its active NT fragment in wild-type neutrophils.^275^ Another study has shown that wild-type and *Gsdmd*^−/^^−^ mice exhibit comparable NET formation kinetics and magnitude in response to NET-inducing agonists, suggesting that this process is independent of cell death. Furthermore, even when canonical inflammasome activation leads to GSDMD cleavage and NET production, NET release does not require caspase or GSDMD participation.^274^ The initial support for GSDMD’s role in NETosis was partly based on the use of the chemical inhibitor LDC7559, which was reported to block both NETosis and GSDMD.^271^ However, a subsequent study revealed that LDC7559’s inhibitory effect on NETosis is due to off-target effects, including the direct inhibition of NADPH oxidase-driven ROS production, rather than specific GSDMD blockade.^276^ This re-evaluation of a key pharmacological tool weakened the original argument for a universal role of GSDMD in NETosis. This ongoing controversy highlights the complex and potentially diverse nature of the NETosis pathway itself. Further in-depth research is still needed to fully understand the specific role of GSDMs.

### Gasdermins in PANoptosis

PANoptosis is not simply the simultaneous occurrence of independent death pathways but a distinct, regulated process controlled by the assembly of a PANoptosome.^277–282^ The PANoptosome is a large, dynamic, and heterogeneous protein complex whose composition is tailored to the specific initiating trigger, such as infection with influenza A virus (IAV), herpes simplex virus 1 (HSV1), or exposure to certain cytokine combinations.^251,278,279,281^ While the precise architecture of the PANoptosome is still being elucidated and is a topic of ongoing research, Z-DNA-binding protein 1 (ZBP1), absent in melanoma 2 (AIM2), RIPK1 and NLRP12 have been identified as sensors (Fig. 2d).^83,283–285^ Once the PANoptosome assembles, it activates downstream effectors leading to different forms of cell death: caspase-3/7 for apoptosis, GSDMD and GSDME for pyroptosis, and RIPK3-MLKL for necroptosis.^277–279,283^ If a pathogen evolves a strategy to inhibit one branch of the cell death response, the PANoptosome can still execute cell death via the remaining, unblocked pathway, ensuring the elimination of the infected cell. Within the PANoptosis framework, GSDMD acts in concert with the other terminal executioners, MLKL and caspase-3/GSDME, to ensure swift and complete dismantling of the cell (Fig. 2d). The combined action of multiple distinct pore-forming proteins (GSDMs and MLKL) likely results in a more rapid and catastrophic loss of membrane integrity than any single pathway alone.^286^ Instead of isolated processes, these pathways are intricately linked. When a strong stimulus is present, the corresponding cell death program usually takes over, and other pathways might be partially suppressed. However, if a key molecule in one cell death pathway is blocked, other pathways can step in and compensate. For instance, in cells deficient in caspase-8 or its adapter, FADD and ZBP1 are upregulated. This triggers the activation of RIPK3 and subsequent MLKL phosphorylation, ultimately driving necroptosis.^287^ Similarly, during *Yersinia* infection, the genetic deletion of RIPK1 abrogates the induction of pyroptosis and apoptosis but concurrently enhances necroptosis.^288^ This has profound therapeutic implications, suggesting that targeting a single executioner molecule might be insufficient to prevent cell death and inflammation in diseases driven by PANoptosis. Effective therapeutic strategies may therefore require targeting upstream nodes of PANoptosome assembly or simultaneously inhibiting multiple downstream executioner pathways.

## Gasdermin-mediated pyroptosis in diseases

Gasdermin-mediated pyroptosis has been extensively investigated in infectious diseases, both bacterial and viral.^289–291^ It is also consistently linked to inflammatory diseases, including cardiovascular disorders, liver diseases and neurodegeneration.^292–294^ In the context of cancer, pyroptosis suppresses tumorigenesis by triggering immunogenic cell death (ICD) and activating cytotoxic lymphocyte infiltration, yet conversely, chronic pyroptotic signaling within the tumor microenvironment (TME) can remodel the stroma to favor immunosuppression and metastasis.^295–297^

### Gasdermins in infectious diseases

#### Bacterial infection

GSDM-mediated pyroptosis plays a crucial role in host defense against infection by inhibiting bacterial replication and releasing inflammatory cytokines and DAMPs to alert the immune system. This pathway is activated by various types of bacterial pathogens, ranging from those causing acute infection with host hyperinflammation (including *Shigella*, *Yersinia*, and *Streptococcus*) to those capable of establishing chronic infections with persistent host inflammatory responses (including *Salmonella*, *Mycobacterium tuberculosis*, and *Helicobacter pylori*).^289,298^ Bacterial pathogens have also evolved to manipulate pyroptosis to evade host immune clearance and promote infection. Although bacterial pathogens have been shown to regulate multiple inflammasome components through diverse mechanisms, this section focuses specifically on their crosstalk with GSDMs.

**Fig. 3:**
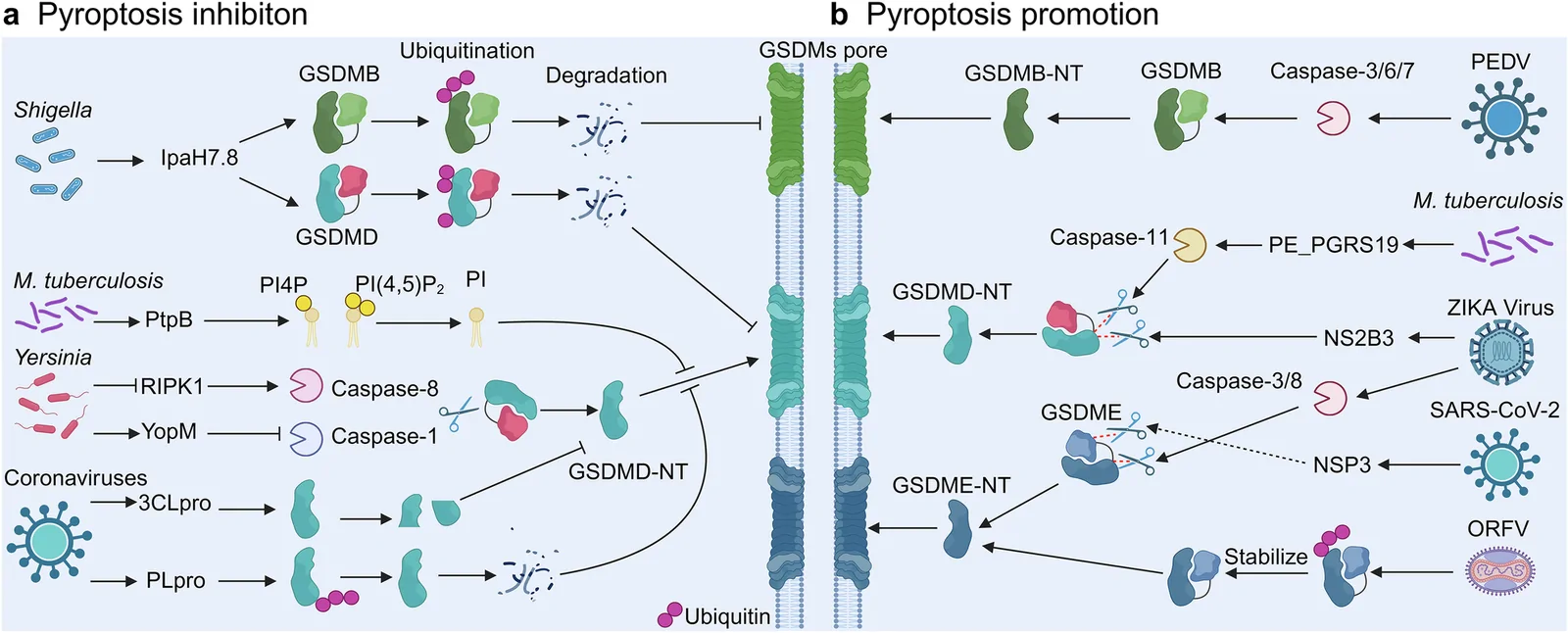
Gasdermin-mediated pyroptosis in infectious diseases. **a** Pathogen-mediated inhibition of pyroptosis. *Shigella* IpaH7.8 promotes the ubiquitination and subsequent proteasomal degradation of GSDMB and GSDMD. The *Mycobacterium tuberculosis* effector PtpB dephosphorylates membrane phosphatidylinositol-4-monophosphate (PI4P) and phosphatidylinositol-(4,5)-bisphosphate (PI(4,5)P_2_), disrupting GSDMD-NT membrane localization and preventing pore formation. *Yersinia* infection depletes glucose, which inhibits RIPK1 and caspase-8 activation, while the *Yersinia* effector YopM inhibits caspase-1 to block GSDMD cleavage. In coronaviruses (SARS-CoV-2, MERS, PEDV, and PDCoV), 3C-like proteases cleave GSDMD at Gln193 to generate inactive fragments. Additionally, papain-like proteases (PLpro) from SARS-CoV-2, PDCoV, and PEDV deubiquitinate GSDMD-NT, promoting its degradation. **b** Pathogen-mediated activation of pyroptosis. PEDV triggers caspase-3/6/7-mediated cleavage of porcine GSDMB. The *M. tuberculosis* effector PE_PGRS19 enhances caspase-11-dependent GSDMD cleavage. Zika virus (ZIKV) protease NS2B3 directly cleaves human GSDMD at Arg249 to release a pore-forming fragment. ZIKV also activates caspase-8, leading to caspase-3-mediated GSDME cleavage. Similarly, the SARS-CoV-2 protease NSP3 cleaves GSDME at Gly370. Orf virus (ORFV) infection enhances pyroptosis by promoting GSDME ubiquitination levels. This figure is created in BioRender

The type III secretion system (T3SS), present in gram-negative bacteria, including *Shigella*, *Yersinia*, and *Salmonella*, and the type VII secretion system (T7SS), present in *Mycobacterium tuberculosis*, can directly activate inflammasomes and induce pyroptosis. Moreover, effector proteins of these pathogens also participate in regulating pyroptosis. While some are detected by host sensors to activate inflammasomes, others degrade critical proteins or block specific pathways to inhibit pyroptosis. For example, the *Yersinia* effector protein YopJ inhibits TAK1 kinase activity to activate caspase-8 for GSDMD cleavage, while conversely, YopM inhibits caspase-1 activity to prevent GSDMD cleavage (Fig. 3a).^21,127,299^ Additionally, *Yersinia* infection causes glucose depletion that activates AMPK, which phosphorylates RIPK1 at Ser321 to suppress host pyroptosis and promote bacterial survival (Fig. 3a).^300^ The *Shigella flexneri* effector OspC3 mediates ADP-riboxanation of caspase-4 and caspase-11, thereby blocking their auto-processing and GSDMD cleavage.^301,302^ The *Shigella* effector IpaH7.8 has also been shown to promote the degradation of human GSDMD by ubiquitination but not mouse GSDMD, indicating that *Shigella* evolved strategies specifically to infect humans.^232^ Furthermore, IpaH7.8 ubiquitinates and targets GSDMB for 26S proteasomal degradation, which protects *Shigella* from the bactericidal activity of natural killer cells by suppressing GZMA-mediated activation of GSDMB (Fig. 3a).^146^ The *Mycobacterium tuberculosis* effector PE_PGRS19 promotes the cleavage of GSDMD by caspase-11 (Fig. 3b).^303^ Conversely, PtpB dephosphorylates phosphatidylinositol-4-monophosphate (PI4P) and phosphatidylinositol-(4,5)-bisphosphate (PI(4,5)P_2_) in the host cell membrane, which disrupts the membrane localization of GSDMD-NT, thereby inhibiting cytokine release and macrophage pyroptosis (Fig. 3a).^304^

Recent studies also indicate that pathogens can exploit pyroptosis to enhance infection. Varela et al. demonstrated that while the canonical Caspb-ASC-IL-1β axis restricts bacterial proliferation, the non-canonical Caspa-Gsdmeb axis actively drives bacterial dissemination by inducing pyroptosis.^305,306^ Additionally, Beckwith et al. used live-cell imaging to reveal that approximately 25% of *M. tuberculosis*-infected macrophages undergo pyroptosis driven by plasma membrane damage. This process facilitates the release of intracellular bacteria, suggesting that pyroptosis may function as a mechanism to promote bacterial dissemination rather than host protection.^307^ Ashida et al. highlighted the dual role of pyroptosis during *Shigella* infection, which is dependent on cell type. In epithelial cells, pyroptosis functions as an antibacterial defense by inhibiting intracellular replication. Conversely, in macrophages, bacteria exploit this pathway to induce cell death, thereby facilitating bacterial release and subsequent dissemination.^308^ Taken together, GSDM-mediated pyroptosis not only serves as a host defense to limit infection but can also be exploited by pathogens to facilitate replication and survival. Although this process is co-regulated by both host and bacterial factors, the precise mechanisms remain to be fully elucidated.

#### Viral infection

Although widely investigated in bacterial infections, GSDM-mediated pyroptosis has recently been implicated in viral infections as well. Many viruses, including herpes simplex virus 1 (HSV-1), murine cytomegalovirus (mCMV), encephalomyocarditis virus (EMCV), West Nile virus (WNV), and severe fever with thrombocytopenia syndrome virus (SFTSV), are known to activate or evade inflammasomes, yet their direct relationship with pyroptosis remains to be fully elucidated.^309–314^ In the case of rotavirus, the RNA helicase DHX9 complexes with NLRP9b to detect short dsRNA segments, activating caspase-1 and GSDMD. This triggers the pyroptosis of infected enterocytes, causing their extrusion into the gut lumen and preventing viral spread to the lamina propria.^97^ Rabies virus (RABV) infection induces pyroptosis in neuronal cells.^315^ Viruses also utilize distinct strategies to evade this pathway. The SARS-CoV-2 nucleocapsid protein directly interacts with GSDMD and hinders GSDMD processing by caspase-1.^316^ During the early stages of infection, RSV upregulates c-FLIP via the PI3K-Akt pathway to block extrinsic apoptosis. Simultaneously, it degrades ZDHHC9 to inhibit GSDMD-mediated pyroptosis, thereby preserving host cell viability for viral replication.^317^ Additionally, the 3C-like proteases of several coronaviruses (SARS-CoV-2, MERS, PEDV, and PDCoV) and enteroviruses (EV71) cleave GSDMD at Gln193, inhibiting pyroptosis and promoting viral replication (Fig. 3a).^318,319^ Furthermore, the 3C-like protease of SARS-CoV-2 has also been shown to cleave GSDMD at additional sites, including Gln29 and His270, which blocks pore formation and pyroptosis.^320^ Similarly, ASFV pS273R cleaves GSDMD at Gly107 to inhibit pyroptosis.^321^ The papain-like protease (PLP) of SARS-CoV-2, PDCoV, and PEDV deubiquitinates GSDMD-NT and promotes its degradation, thereby inhibiting pyroptosis (Fig. 3a).^322^

However, in other contexts, viruses exploit pyroptosis to exacerbate host damage and accelerate disease pathogenesis. Evidence suggests that in severe acute infections involving SARS-CoV-2, influenza A virus (IAV), and norovirus, GSDMD-driven pyroptosis frequently exacerbates disease severity. For example, in COVID-19, excessive pyroptosis in alveolar macrophages triggers cytokine storms and tissue damage rather than contributing to effective viral control.^323^ In placental cells of pregnant mice, the cytosolic sensor RIG-I recognizes Zika virus (ZIKV) RNA to activate caspase-8, which subsequently triggers caspase-3-mediated GSDME cleavage and pyroptosis, leading to fetal abnormalities or miscarriage (Fig. 3b).^324^ Additionally, NS2B3 of ZIKV can directly cleave human GSDMD at Arg249 to release an NT fragment to induce pyroptosis (Fig. 3b).^325^ EV71 infection activates caspase-3 to cleave GSDME and induce pyroptosis, which is important for pathogenesis.^326^ Similar mechanisms are observed in IAV H7N9 and H1N1.^327,328^ A recent study also reported that GSDMD amplifies influenza virus-induced mortality through neutrophil amplification of inflammation.^329^ Foot-and-Mouth Disease Virus (FMDV) 3C protease cleaves porcine GSDME at Gln271 to trigger pyroptosis, and the SARS-CoV-2 protease NSP3 cleaves GSDME at Gly370 to release a pore-forming fragment that contributes to COVID-19 immunopathology.^330,331^ Complex regulatory patterns are also observed in EV-D68 infection, where the viral 3C protease inactivates GSDMD, while the 2A protease activates GSDME.^331^ PEDV infection has also been shown to cleave GSDMB to induce pyroptosis (Fig. 3b).^147^ Furthermore, Orf virus (ORFV) infection promotes pyroptosis by reducing GSDME ubiquitination levels (Fig. 3b).^332^ In chronic infections such as HIV, CD4⁺ T-cell depletion in lymphoid tissues is now attributed primarily to GSDMD-mediated pyroptosis rather than apoptosis. This process drives chronic inflammation via IL-1β secretion, creating a vicious cycle of persistent inflammation and immunodeficiency.^333^ Concurrently, GSDMD-driven pyroptosis in gut mucosal-associated invariant T (MAIT) cells of HIV-infected individuals promotes inflammatory cytokine release and hinders immune reconstitution.^334^ Collectively, these findings indicate that the ultimate effect of pyroptosis is determined by the specific pathogen, cell type, and infection stage. Therefore, developing precision therapeutic strategies requires a systematic evaluation of GSDM dynamics to distinguish between protective and deleterious functions, enabling the design of pathway-specific interventions that mitigate immunopathology without compromising essential host defense mechanisms.

### Gasdermins in non-infectious inflammatory diseases

Beyond bacterial and viral infections, GSDMs have been investigated in various non-infectious inflammatory diseases (Table 3). Here, we discuss these functions using a systematic classification.

**Table 3:** Pyroptosis in non-infectious diseases

Gasdermin	Disease context	Target cell type	Functional annotation/pathological effect	Refs
GSDMA	Alopecia/Skin inflammation	Hair follicles/Keratinocytes	Causes constitutive activation, aberrant hair follicle differentiation, and skin inflammation.	^16,17,38^
GSDMB	Inflammatory Bowel Disease (IBD)	Intestinal epithelial cells (IECs)	Promotes barrier restoration (PDGF-A-FAK axis) or exacerbates injury via the unfolded protein response, depending on isoform expression and activation context.	^37,341^
	Asthma	Bronchial epithelial cells (BECs)	Upregulation correlates with increased disease susceptibility and airway damage	^346,347^
GSDMC	Psoriasis/Atopic Dermatitis	Epidermal keratinocytes	Reported to be enriched in lesions (its functional role in disease progression remains to be fully defined)	^403^
	IBD (UC/Crohn’s)	IECs	Promotes epithelial damage and inflammation during helminth infection or IL-10 deficiency	^342,343^
GSDMD	Acute Pancreatitis	Acinar cells/Macrophages	Protects acinar cells locally, but contributes to systemic pathogenesis, likely through macrophage activation, and is associated with enhanced Th1/Th17 responses	^335^
	IBD (UC/Crohn’s)	IECs/Macrophages	Pathogenic in IECs (lytic death/goblet cell loss), but protective in macrophages (restrains cGAS-mediated inflammation)	^336–339^
	Asthma/COPD/Acute Lung Injury	BECs/Alveolar macrophages/Endothelial cells	Impairs mucociliary clearance, promotes excessive inflammatory cytokine release, and drives pulmonary edema and alveolar wall thickening	^349–352^
	Atherosclerosis/Myocardial Infarction	Macrophages/Neutrophils	Exacerbates foam cell formation and drives detrimental immunopathology via excessive neutrophil mobilization from bone marrow	^354,355,358,362^
	Diabetic Kidney Disease/AKI	Podocytes/Tubular cells/Glomerular endothelial cells	Induces podocyte destruction, tubular injury, and glomerular endothelial dysfunction via NETs; Alternatively, may exert a protective role in AKI by inhibiting tubular necroptosis	^366,368–372,374,375^
	Alzheimer’s disease (AD)/Parkinson’s disease (PD)	Microglia/Astrocytes	Activated by Aβ, hyperphosphorylated Tau, or α-synuclein to drive neuroinflammation, T cell infiltration, and neuronal death	^380–384,392,393^
	Neuroinflammation/Epilepsy (TLE)	Brain endothelial cells/Microglia	LPS-primed activation mediates BBB breakdown in brain endothelial cells; Conversely, exerts a protective role in TLE by maintaining microglial phagocytic activity	^399,400^
	SLE/Systemic Sclerosis	Neutrophils/Macrophages	Required for neutrophil lytic cell death and DNA release; Promotes macrophage-driven skin fibrosis and autoantibody production	^409–413^
	Rheumatoid Arthritis/Osteoarthritis	Fibroblast-like synoviocytes/Chondrocytes	Induces synovial inflammation, cartilage degeneration, and promotes fibrosis	^416,420–422^
GSDME (DFNA5)	Crohn’s Disease	IECs	Converts TNF-α-induced cell shedding into pyroptotic cell death, releasing intracellular contents to drive mucosal inflammation	^344,345^
	Atherosclerosis	Macrophages	Promotes ox-LDL-induced inflammation and pyroptosis, thereby driving atherosclerotic plaque progression	^356^
	AKI/Obstructive nephropathy	Renal tubular cells	Exacerbates ischemia-reperfusion injury and induces hydronephrosis and renal fibrosis by releasing HMGB1	^367–369,378^
	Neurodegeneration (AD/PD/ALS)	Neurons/Axons	The active N-terminal fragment may impair mitochondrial function in neurons, contributing to neurodegeneration	^380–383,395,398^
	Stroke/HIV-associated neurocognitive disorder	Endothelial cells/Neurons	Induces blood-brain barrier disruption during ischemia; Causes HIV-associated neurocognitive disorder via neuronal pyroptosis	^396,397^
	Rheumatoid Arthritis	Monocytes/Fibroblast-like synoviocytes	Activated by TNF or NETs to drive joint inflammation and rheumatoid arthritis pathogenesis	^414,415^

#### Gastrointestinal inflammation

**Fig. 4:**
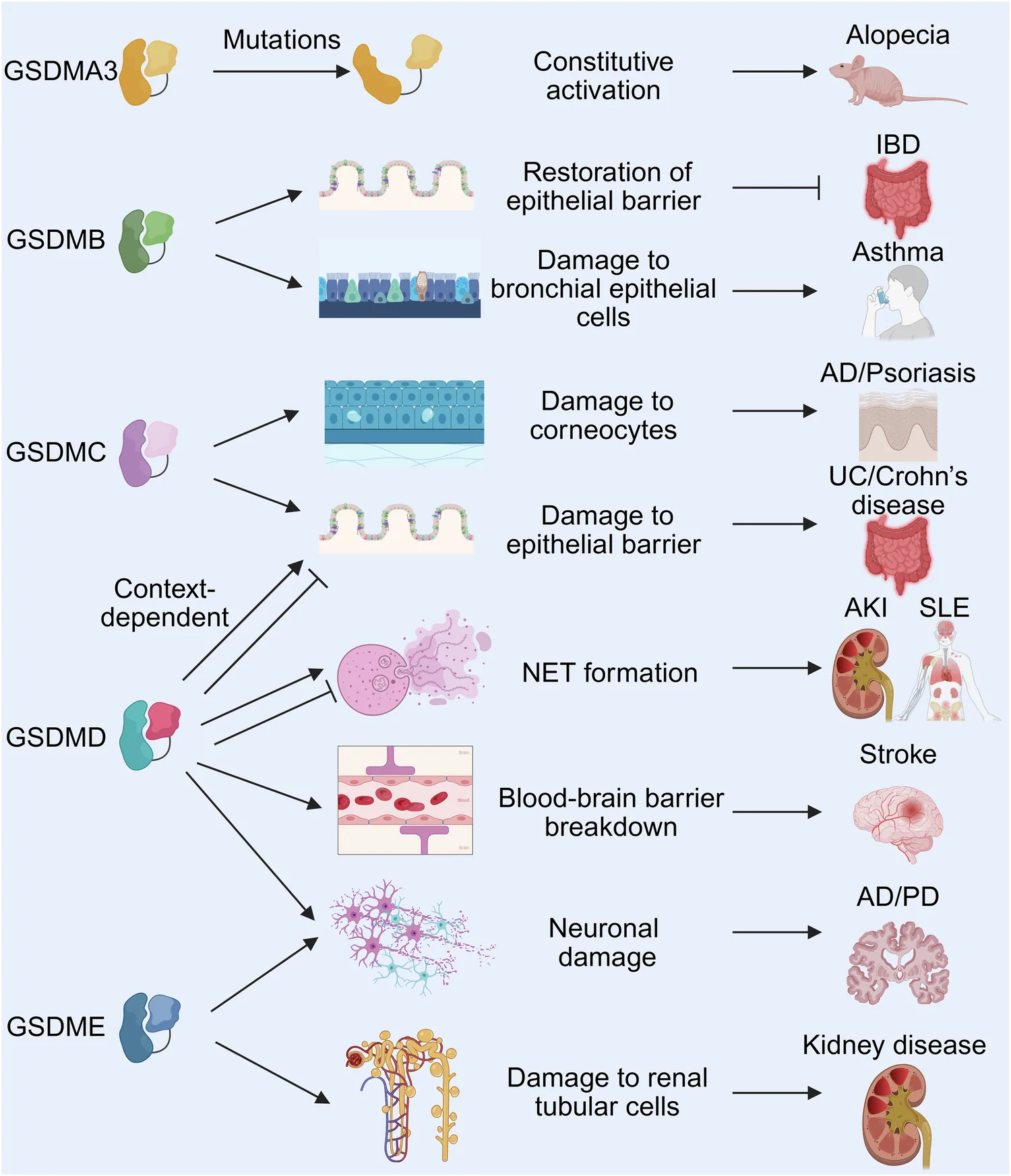
Gasdermin-mediated pyroptosis in inflammatory diseases. Mutations in mouse *Gsdma3* that cause premature truncation or loss of domain binding lead to constitutive activation and alopecia. GSDMB plays a critical role in restoring epithelial barrier function and resolving inflammation. Furthermore, upregulated GSDMB levels in bronchial epithelial cells (BECs) of asthmatic patients correlate with increased disease susceptibility. GSDMC is enriched in corneocytes, where it drives pyroptosis to promote psoriasis and atopic dermatitis. GSDMC is also significantly upregulated in ulcerative colitis (UC) and Crohn’s disease. The role of GSDMD in UC and Crohn’s disease appears context dependent. While generally considered a driver of mucosal injury, GSDMD may also regulate inflammation during bacterial invasion. GSDMD-dependent NET formation contributes to acute kidney injury (AKI) and systemic lupus erythematosus (SLE). In the nervous system, GSDMD-mediated pyroptosis disrupts the blood‒brain barrier (BBB) during stroke, while GSDMD and GSDME promote neuronal degradation in Alzheimer’s disease (AD) and Parkinson’s disease (PD). Finally, TNF-α triggers caspase-3-mediated GSDME cleavage in renal tubular cells, causing obstructive nephropathy. This figure is created in BioRender

The function of GSDMs extends to inflammatory disorders of the gastrointestinal tract and its associated digestive organs, including the pancreas and liver. In acute pancreatitis (AP), GSDMD plays a paradoxical role. While it protects acinar cells by inhibiting AKT/mTOR-mediated enzyme synthesis and inducing MUC1 barriers, these local benefits are outweighed by systemic pathogenic effects, including macrophage activation and Th1/Th17 expansion. Consequently, GSDMD knockout alleviates AP, identifying it as a viable therapeutic target despite its complex dual functions.^335^ The role of GSDMD in ulcerative colitis (UC) remains a subject of debate, characterized by two distinct mechanistic paradigms.^336–338^ The prevailing perspective identifies GSDMD as a primary driver of mucosal injury. Research by Bulek et al. and Xiao et al. demonstrates that GSDM-mediated pore formation in intestinal epithelial cells (IECs) triggers cytokine release and lytic cell death, thereby disrupting physical barrier function. Supporting this view, GSDMD- and GSDME-deficient mice exhibit reduced disease severity in DSS-induced colitis models.^336,337^ In contrast, Ma et al. suggested that GSDMD within colonic macrophages regulates cGAS-mediated inflammation in response to bacterial invasion or epithelial damage, thereby providing protection against colitis development.^339^ While this research suggests a protective function independent of the microbiota, Gao et al. revealed that GSDMD expression specifically in IECs, rather than infiltrating immune cells, is critical for colitis progression. They found that dysregulated overgrowth of commensal *Escherichia coli* during colitis mediates GSDMD activation. Furthermore, activated GSDMD promotes the release of IL-18 without affecting its transcription or maturation levels, which subsequently mediates goblet cell loss to drive colitis pathology.^338^ Beyond GSDMD, other gasdermin family members have been reported to play crucial roles in inflammatory bowel disease (IBD).^340^ GSDMB has been identified as a critical factor for the restoration of epithelial barrier function and the resolution of inflammation (Fig. 4).^37^ Conversely, epithelial GSDMB is also reported to modulate the hyperactive unfolded protein response in IECs by upregulating BHLHA15, thereby mediating intestinal barrier injury.^341^ A recent transcriptome analysis of hundreds of intestinal biopsies found that *GSDMC* is the only gasdermin gene showing significant upregulation in both ulcerative colitis and Crohn’s disease patients.^342^ In murine models, GSDMC cleavage is observed in both DSS-induced and IL-10 deficiency-induced colitis. Notably, the deletion of murine GSDMC attenuates DSS-induced colitis exacerbated by helminth infections or IL-10 deficiency, suggesting that cytokine release resulting from GSDMC pore formation drives inflammation (Fig. 4).^343^ GSDME cleavage is also observed in the inflamed colonic mucosa of patients with Crohn’s disease. GSDME-mediated epithelial cell pyroptosis drives intestinal inflammation through the release of proinflammatory intracellular contents.^344^ Other research also indicates that under inflammatory conditions, caspase-3 cleaves GSDME in IECs, effectively converting TNFα-induced cell shedding into a pyroptotic cell death process.^345^ The function of GSDMA in the intestine remains poorly characterized. Intestinal GSDMA appears to be expressed exclusively by enteroendocrine cells (EECs) and goblet cells, both of which contribute critically to barrier function and mucosal homeostasis. Taken together, the role of GSDMs in IBD is complex and context dependent, governed by the microenvironment, dominant pathogenic signals, specific cell types, and microbiota status. Future research must clarify the activation sources and effector cells of GSDMD across different IBD subtypes. Additionally, efforts should focus on developing cell-specific GSDMD regulatory strategies and integrating metagenomics with host transcriptomics to achieve precise treatment.

#### Respiratory inflammation

Research on GSDMs in respiratory diseases is increasingly focusing on chronic airway inflammation and acute lung injury (ALI). The activation of GSDMs in airway epithelial cells serves as a critical driver for the progression of asthma and chronic obstructive pulmonary disease (COPD).^294^ Upon exposure to environmental allergens, GSDMD-mediated pyroptosis in bronchial epithelial cells (BECs) induces severe airway inflammation. The resulting damage to the epithelium impairs mucociliary clearance, allowing various allergens to infiltrate lung tissues. Additionally, elevated GSDMB levels are observed in asthmatic patients, where upregulated expression in BECs correlates with increased disease susceptibility (Fig. 4).^346,347^ Furthermore, pyroptosis in macrophages acts as a significant promoter of asthma development.^348^ For instance, perfluoroalkyl substances (PFAS) have been reported to induce GSDMD-dependent pyroptosis in macrophages, which aggravates asthmatic inflammation by promoting the secretion of IL-4 and IL-1β.^349^ Moreover, caspase-4/11-mediated GSDMD cleavage has been identified as a critical contributor to the pathology of allergic airway inflammation.^350^ Cigarette smoke induces GSDMD-mediated pyroptosis, which serves as a critical driver of COPD. Melatonin has been shown to significantly attenuate these CSE-induced effects, offering potential protection against COPD.^351^

Persistent, excessive inflammation driven by alveolar macrophages (AMs) is critical to the pathogenesis of acute lung injury (ALI), with AM pyroptosis playing a central role. It has been reported that pyroptosis in AMs is activated through the NLRP3/caspase-1/GSDMD axis. This activation leads to severe lung injury, characterized by the infiltration of inflammatory cells, pulmonary interstitial edema, hemorrhage, and alveolar wall thickening. Consequently, pharmacological agents targeting this pyroptotic pathway are currently being explored as promising therapeutic strategies for ALI.^294^ Furthermore, pyroptosis in pulmonary endothelial cells is also a critical contributor to ALI. Research indicates that caspase-11/GSDMD-mediated pyroptosis in these cells acts as a primary driver of pulmonary edema. The endothelial-specific deletion of caspase-11 has been shown to attenuate pulmonary inflammation and mitigate lung damage.^352^ The involvement of other GSDM family members in respiratory inflammatory diseases remains to be fully explored. Collectively, these findings underscore the significance of GSDM-mediated pyroptosis in respiratory pathology. While emerging therapeutic agents show promise in preclinical models, future research must focus on developing potent inhibitors to intervene effectively in clinical settings.

#### Cardiovascular inflammation

Emerging evidence highlights that GSDM-mediated pyroptosis is a critical driver in the pathogenesis of both chronic and acute cardiovascular diseases, including atherosclerosis, myocardial infarction, and abdominal aortic aneurysm (AAA).

Atherosclerosis is characterized by the abnormal deposition of lipids and lipoproteins within arterial walls, accompanied by the accumulation of foam cells derived from infiltrating monocytes and trans-differentiated vascular smooth muscle cells (VSMCs). Inflammation drives this pathology through the sustained recruitment of monocytes into plaques.^353^ GSDMs have emerged as key mediators in this process. Research indicates that GSDMD exacerbates foam cell formation in an IL-1β-dependent manner, whereas GSDMD-deficient macrophages remain resistant to NLRP3 inflammasome-mediated reductions in cholesterol efflux. In LDLr antisense oligonucleotide (ASO)-induced hyperlipidemic mice, GSDMD deficiency significantly reduced atherosclerotic lesion areas and leukocyte infiltration while shifting cell death from pyroptosis to apoptosis. Consistent with these findings, cleaved GSDMD has been identified in macrophage- and smooth muscle cell-rich regions of human plaques.^354^ Additionally, GSDMD-mediated pyroptosis activated by caspase-11 in macrophages has also been reported to promote the pathogenesis of atherosclerosis.^355^ Similarly, the ablation of GSDME in macrophages represses ox-LDL-induced inflammation and pyroptosis, thereby inhibiting the overall progression of atherosclerosis.^356^

Myocardial infarction (MI) is increasingly linked to GSDM-mediated pyroptosis.^357^ In the early stages of acute myocardial infarction (AMI), GSDMD cleavage is observed in activated leukocytes. Studies show that GSDMD deficiency significantly reduces infarct size, improves cardiac function, and increases post-AMI survival. Mechanistically, this protection is attributed to the suppression of excessive, GSDMD-dependent neutrophil production and mobilization from the bone marrow (typically peaking 24 h post-AMI), which otherwise drives detrimental immunopathology.^358^ Additionally, the GSDMD expression level in peripheral blood mononuclear cells is also elevated, which is closely associated with the pyroptosis of AMI and increases the risk of major adverse cardiovascular events after AMI.^359^ Pyroptosis signaling is notably activated within 24 h of AMI, and treatment with VX-765, a caspase-1 inhibitor, has been shown to significantly reduce infarct size and preserve cardiomyocyte function.^360^ Furthermore, GSDMD is markedly elevated in the plasma of mice during early AMI and in the supernatant of hypoxia-exposed cardiomyocytes in a time-dependent manner. Notably, treatment with MCC950 and VX-765 effectively decreases hypoxia-induced cardiomyocyte injury while mitigating oxidative stress and inflammation.^361^ Supporting this inflammatory mechanism, another study indicated that MI promotes the accumulation of inflammasome-primed neutrophils in the bone marrow. These primed neutrophils secrete IL-1β via GSDMD pores to drive granulopoiesis, resulting in the excessive production and infiltration of neutrophils into the ischemic heart.^362^ In the context of thrombotic microangiopathy (TMA), GSDMD plays a pathogenic role. Notably, GSDMD deficiency or inhibition by disulfiram has been shown to suppress neutrophil pyroptosis and NET formation, ultimately mitigating immunothrombosis.^363^ In summary, pyroptosis exacerbates cardiovascular pathogenesis by amplifying inflammation in response to various stress signals, including hypoxia and metabolic stress. However, the involvement of diverse cell types and complex networks of local and distal cells adds significant complexity to these outcomes. Consequently, the development of specific small-molecule inhibitors targeting pore formation and upstream pathways is urgently needed to dampen severe inflammation in chronic cardiovascular diseases and reduce mortality associated with acute cardiovascular events.

#### Renal inflammation

Studies have claimed that pyroptosis is a pathogenic mechanism in renal diseases such as acute kidney injury and diabetic kidney disease (DKD).^364,365^

In the context of ischemia‒reperfusion injury-induced AKI (IRI-AKI), research has found that caspase-11-mediated GSDMD cleavage promotes the release of IL-18 in tubular cells, driving renal functional deterioration.^366^ Similarly, caspase-3-mediated GSDME cleavage has been shown to exacerbate ischemia‒reperfusion injury.^367^ Beyond canonical cleavage pathways, ER stress-induced CHOP activation exacerbates renal injury by promoting both GSDMD and GSDME cleavage while simultaneously upregulating GSDME transcription.^368,369^ However, conflicting evidence suggests that GSDMD may exert a non-cell autonomous protective role in the tubular compartment during IRI. Rather than inducing damage via direct pyroptosis of renal tubular epithelial cells, GSDMD appears to regulate the peritubular microenvironment specifically by inhibiting necroptosis, thereby preserving renal tubules from MLKL-dependent necroptotic injury.^370^ The precise reasons underlying these discrepancies remain to be determined.

In DKD, pyroptosis has been observed in podocytes, glomerular endothelial cells (GECs) and tubular epithelial cells.^371–373^ Under hyperglycemic conditions, the caspase-4/11-GSDMD axis-mediated pyroptosis pathway is activated in podocytes. This activation leads to structural destruction, loss of function, and the release of pro-inflammatory factors, thereby driving the onset and progression of DKD.^371^ Increased NET deposition is observed in the glomeruli of both DKD patients and diabetic mice. These NETs induce pyroptosis in GECs, thereby promoting GEC injury and subsequent glomerulopathy. Notably, strengthening the GEC surface charge using oleylamine significantly inhibits this NET-induced pyroptosis (Fig. 4).^372^ Furthermore, the non-canonical caspase-11/GSDMD pathway has been identified as a specific driver of GEC pyroptosis, leading to severe endothelial injury and accelerating the progression of diabetic nephropathy.^374^ In type 2 diabetic kidney disease (T2DKD), TLR4 activation promotes the release of inflammatory cytokines and induces pyroptosis in renal tubular epithelial cells via the canonical caspase-1/GSDMD pathway. The upregulation of GSDMD promotes pyroptosis while simultaneously inhibiting apoptosis.^373^ Hyperglycemic conditions also upregulate alpha-kinase 1 (ALPK1), triggering caspase-1/GSDMD-mediated pyroptosis. This process drives the renal tubular injury and interstitial inflammation characteristic of diabetic nephropathy.^375^ Hyperuricemic conditions lead to the cleavage of GSDMD by both caspase-1 and caspase-11, thereby promoting the progression of hyperuricemic nephropathy. Furthermore, this GSDMD activation induces NET formation, which subsequently drives renal fibrosis in the context of the disease.^376,377^

In obstructive nephropathy, TNF-α release triggers the activation of caspase-3, which subsequently cleaves GSDME to induce pyroptosis in renal tubular cells. The resulting release of HMGB1 then contributes to the late-stage progression of hydronephrosis, inflammation, and fibrosis.^378^ Another study demonstrated that unilateral ureteral obstruction (UUO) induces caspase-11-mediated GSDMD activation in neutrophils, resulting in excessive NET formation. These GSDMD-dependent NETs trigger nuclear translocation of p65 in macrophages, which subsequently boosts the production of inflammatory cytokines. Furthermore, this signaling cascade activates the TGF-β1/Smad pathway, driving α-SMA expression and macrophage-to-myofibroblast transition (MMT), ultimately promoting the development of renal fibrosis (Fig. 4).^379^

Taken together, GSMD-mediated pyroptosis, especially GSDMD and GSDME, is crucial in the pathogenesis of kidney diseases. Future research efforts should accelerate the development of specific pyroptosis inhibitors alongside the advancement of gene therapy strategies, thereby driving the translation of mechanistic insights into effective clinical applications.

#### Neuroinflammation

GSDMs have been shown to play critical roles in neuroinflammatory diseases, such as Alzheimer’s disease (AD) and Parkinson’s disease (PD). In the context of AD, the main pathological markers Amyloid-β (Aβ) and hyperphosphorylated Tau protein have been shown to directly trigger pyroptosis pathways. Aβ activates the NLRP3 inflammasome in microglia and astrocytes, promoting caspase-1 activation and GSDMD cleavage. Hyperphosphorylated Tau has been demonstrated to activate caspase-1 or caspase-3, thereby mediating neuronal pyroptosis via GSDMD or GSDME.^380–383^ Recent research has demonstrated that caspase activates GSDMD in the spleen earlier than in the brain during disease progression. Myeloid GSDMD deficiency hinders peripheral T-cell activation and subsequent brain infiltration, dampening neuroinflammation. This process involves the upregulation of T-cell PD-1 via the IL-1β/NF-κB axis, which suppresses regulatory T cells. Therapeutically, dual inhibition of GSDMD and PD-1 attenuates AD pathology.^384^ Another study demonstrated that in AD, Drp1 hyperactivation in mature oligodendrocytes induces mitochondrial fragmentation and HK1-mediated glycolytic defects. This metabolic stress activates caspase-1/GSDMD-dependent pyroptosis, thereby driving demyelination, white matter loss, and subsequent cognitive deficits.^385^ Pharmacological interventions targeting the pyroptosis pathway have demonstrated significant therapeutic potential. For instance, administration of the caspase-1 inhibitor VX-765 dose-dependently reverses episodic and spatial memory impairments, as well as hyperactivity, in a mouse model of AD.^386^ Similarly, the natural compound Cornuside exerts neuroprotective effects by activating mitophagy. This upstream mechanism suppresses the NLRP3/GSDMD pathway, thereby attenuating neuronal and synaptic damage and ameliorating cognitive deficits (Fig. 4).^387^ These findings highlight the critical involvement of GSDM-mediated pyroptosis in AD pathology, while the precise mechanisms governing this process across distinct cell types remain to be fully elucidated.

Despite the complex etiology, recent studies highlight that neuroinflammation-driven pyroptosis plays an important role in the pathogenesis of PD.^388^ In the substantia nigra and striatum, caspase-1, GSDMD, IL-1β, and IL-18 are significantly upregulated, with GSDMD-N directly implicated in dopaminergic neuronal death.^389–391^ Fibrillar α-synuclein activates TLR2/NF-κB pathway signaling and the NLRP3 inflammasome, driving caspase-1-mediated GSDMD cleavage and cytokine release.^392,393^ In MPTP-induced PD models, caspase-1 knockout mice exhibit reduced microglial activation, enhanced dopaminergic neuron survival, and significant improvements in motor function.^394^ Beyond microglia, astrocytes and infiltrating peripheral immune cells also contribute to the amplification of pyroptosis. Furthermore, dopaminergic neurons themselves may undergo pyroptosis via the caspase-3/GSDME or caspase-1/GSDMD pathways, particularly in advanced disease stages (Fig. 4).^395^ Targeting the pyroptotic pathway, especially the NLRP3/caspase-1/GSDMD axis, offers a promising disease-modifying therapeutic strategy. Thus, interventions aimed at inhibiting this pathway could provide novel avenues for both therapeutic development and diagnostic tools in PD management.

Furthermore, GSDM-mediated pyroptosis has been implicated in other neuroinflammatory diseases. A study indicated that HIV infection of the brain triggers the convergent activation of caspase-1 and caspase-3 by viral protein R (Vpr), which results in GSDME-mediated neuronal pyroptosis, leading to HIV-associated neurocognitive disorder.^396^ In the acute stage of ischemic stroke, hypoxanthine levels are significantly elevated. Mechanistically, hypoxanthine induces intracellular Ca^2+^ overload, which triggers caspase-3-mediated GSDME cleavage and subsequent pyroptosis in endothelial cells. This process ultimately leads to blood‒brain barrier disruption and aggravated brain injury.^397^ In neurologic diseases, neurotoxins and ALS proteins induce caspase-3/7-dependent GSDME cleavage. The active GSDME-N fragment targets axonal mitochondria to cause depolarization and energy collapse. This mitochondrial dysfunction disrupts axonal transport, promoting neurite retraction and permanent neuronal injury.^398^ A recent study demonstrated that LPS stimulation of brain endothelial cells (bECs) primes the expression of caspase-11 and CD14, which induces GSDMD-mediated plasma membrane permeabilization and pyroptosis, resulting in breakdown of the blood‒brain barrier (BBB).^399^ Conversely, GSDMD has been shown to play a protective role in temporal lobe epilepsy (TLE). Specifically, GSDMD knockdown attenuates the phagocytic activity of microglia, leading to the accumulation of apoptotic neurons and exacerbating seizure susceptibility.^400^ These findings suggest that the complete inhibition of GSDMD might be counterproductive, highlighting the need for a cautious and context-dependent assessment of GSDM-targeted therapies.

#### Cutaneous and mucosal inflammation

Apart from the intestinal mucosal inflammation mentioned above, pyroptosis is involved in psoriasis, atopic dermatitis (AD), systemic lupus erythematosus (SLE), and systemic scleroderma (SSc).^401^

Psoriasis is a chronic immune disorder characterized by erythema, papules, and desquamation, in which GSDMD is significantly upregulated. Studies using imiquimod-induced psoriasis-like dermatitis (IIPLD) mice and M5-stimulated keratinocytes revealed increased levels of cleaved caspase-1, GSDMD, and IL-1β, accompanied by typical pyroptotic cell morphology.^402^ Single-cell RNA sequencing analysis further revealed that GSDMA and GSDMC are enriched in the stratum corneum (SC), whereas the stratum basale (SB) exhibits high expression of core pyroptosis genes, including caspase-1, caspase-8, GSDMB, and GSDMD.^403^ Therapeutically, inhibiting GSDMD/GSDME-mediated pyroptosis using agents such as disulfiram and ganoderic acid A, or via genetic knockout, suppresses psoriatic dermatitis in mice, highlighting the potential of this pathway as a treatment target.^402,404–406^ Nevertheless, further research is required to determine which specific factors within the psoriatic immune microenvironment initiate this keratinocyte pyroptosis.

Atopic dermatitis is a chronic, recurrent inflammatory dermatosis characterized by scaly, pruritic, and erythematous lesions situated on the convex skin surface. In the context of AD, scRNA-seq analysis revealed elevated GSDMC and GSDMD expression in lesional keratinocytes. These cells are stratified into GSDMC-high GSDMD-low, GSDMC-high GSDMD-high, and GSDMC-low GSDMD-high subpopulations, with lesion-derived keratinocytes predominantly mapping to the GSDMC-high GSDMD-high subset (Fig. 4).^403^ Therapeutically, inhibiting GSDMD-mediated pyroptosis via disulfiram, matrine, or arctiin blocks keratinocyte cell death and effectively alleviates disease symptoms in AD mouse models.^407,408^

Systemic lupus erythematosus is a systemic autoimmune disorder defined by autoantibody production, immune complex deposition, and widespread organ involvement, notably in the skin, kidneys, and nervous system. GSDMD and IL-1β mRNA are upregulated in PBMCs from SLE patients.^409^ Studies have demonstrated that GSDMD is required for neutrophil lytic cell death and DNA release triggered by SLE serum and IFN-γ. This process is driven by FcγR-mediated downregulation of Serpinb1, which activates caspase-1/11 to cleave GSDMD. Additionally, oxidized mtDNA facilitates GSDMD-NT oligomerization. Therapeutically, targeting this axis proves effective, as both GSDMD deficiency and disulfiram treatment significantly attenuate disease severity.^409,410^ Conversely, another study demonstrated that GSDMD deficiency leads to enhanced mortality, more severe renal and pulmonary inflammation, and exacerbated autoantibody production in response to imiquimod, suggesting that GSDMD may play a previously unappreciated protective role in systemic autoimmunity (Fig. 4).^411^ Consequently, the precise contribution of GSDMs to SLE pathogenesis appears complex and warrants further investigation.

Systemic sclerosis is a rare autoimmune disorder affecting connective tissue that is characterized by fibrosis, inflammation, and vasculopathy. In patients with SSc, pyroptotic and inflammatory proteins, including NLRP3, cleaved caspase-1, GSDMD-NT, and IL-18, are elevated in the serum, while skin macrophages exhibit increased ASC aggregation and GSDMD expression.^412^ GSDMD is significantly upregulated and activated in the skin of both SSc patients and bleomycin-induced mouse models. Notably, GSDMD deficiency ameliorates bleomycin-induced skin fibrosis, reduces macrophage infiltration, and attenuates the inflammatory response.^413^ Treatment with MCC950, disulfiram, or necrosulfonamide has also been found to attenuate cellular and cutaneous fibrosis, thereby alleviating disease symptoms.^412^

Taken together, GSDM-mediated pyroptosis drives disease in psoriasis, atopic dermatitis, SLE, and systemic sclerosis, suggesting therapeutic potential for inhibitors such as disulfiram. However, given GSDMD’s conflicting protective and pathogenic roles in SLE, defining context-specific immune triggers is essential to refine therapeutic strategies.

#### Musculoskeletal and articular inflammation

Despite limited investigation, GSDM-mediated pyroptosis appears to be a key pathogenic driver in musculoskeletal disorders, including rheumatoid arthritis and osteoarthritis.

Rheumatoid arthritis (RA) is a chronic autoimmune disease characterized by persistent synovitis and systemic inflammation, leading to cartilage loss and bone erosion. A study demonstrated that GSDME-NT expression in monocytes from RA patients correlates positively with disease activity. Mechanistically, TNF induces pyroptosis in monocytes and macrophages by activating the caspase-3/GSDME pathway, thereby driving RA pathogenesis. Notably, either caspase-3 inhibition or GSDME deficiency effectively alleviated symptoms in a collagen-induced arthritis (CIA) mouse model.^414^ NETs also promote RA progression by inducing caspase-3/GSDME-mediated pyroptosis in fibroblast-like synoviocytes (FLSs).^415^ Similarly, caspase-1-mediated cleavage of GSDMD induces pyroptosis in FLSs, further promoting pathogenesis.^416^ Additionally, caspase-1 activation is frequent in the lymph node-resident T cells of RA patients due to MRE11A loss of function. This defect causes cytosolic mtDNA leakage, thereby initiating inflammasome assembly and pyroptosis.^417^ Agents such as amiloride and the calcium chelator BAPTA-AM, as well as natural compounds such as punicalagin and the combination of mangiferin and cinnamic acid, have been found to prevent GSDMD cleavage by inhibiting the NLRP3 inflammasome, thereby offering therapeutic potential for RA.^418,419^

Similar to RA, GSDMD-mediated pyroptosis plays a critical role in osteoarthritis (OA). In rat models of knee osteoarthritis (KOA), the mRNA levels of GSDMD and caspase-1 are significantly elevated in synovial tissues, while treatment with the specific caspase-1 inhibitor Ac-YVAD-CMK attenuates inflammatory responses and fibrosis. Knockdown of caspase-1 or GSDMD also suppresses the expression of fibrotic markers in synovial cells.^420^ In the inflammatory microenvironment of the joints, extracellular vesicles secreted by macrophages activate the caspase-4/11-GSDMD pathway in chondrocytes, leading to cartilage degeneration.^421^ In both OA patients and CIOA mice, synovial macrophages undergo GSDMD-mediated pyroptosis and produce ROS, thereby aberrantly activating the mTORC1 pathway and inducing MMP9 expression in the synovium.^422^ Further investigation is required to define GSDM-mediated mechanisms and cell-to-cell crosstalk throughout the progression of musculoskeletal and muscle inflammatory diseases. These insights are critical for the development of novel therapeutic interventions. The regenerative potential of pyroptosis warrants significant attention. Notably, intracellular signals released via caspase-11/GSDMD-mediated pyroptosis stimulate osteoprogenitors and drive osteogenic differentiation in the periosteum.^423^ Consequently, future research should explore whether controlled modulation of this pathway can be harnessed to enhance fracture healing and bone regeneration in clinical settings.

### Gasdermins in pyroptosis-dependent cancer regulation

GSDM-mediated pyroptosis acts as a double-edged sword in cancer. While extensive research demonstrates its ability to boost antitumor immunity, it also drives tumor progression depending on the specific microenvironmental context.^2,3,40,41^

A recombinant fully human IgG1 bispecific antibody, IBI315, was developed to trigger GSDMB-mediated pyroptosis in tumor cells, leading to the activation and recruitment of T cells. Activated T cells secrete IFN-γ, which further upregulates GSDMB expression, establishing a positive feedback loop that amplifies both T-cell activation and tumor cell elimination.^424^ A recent study developed a ROS-responsive liposomal nanoplatform designed to codeliver a polymeric STING agonist and a GSDMB plasmid for dual neuro-immune modulation. Upon tumor-selective activation within metastatic bone niches, this nanotherapy induces STING-driven immune priming alongside GSDMB-mediated pyroptosis, thereby triggering potent antitumor responses.^425^ In colorectal cancer (CRC), hypoxia and low glucose trigger caspase-6-mediated cleavage of GSDMC. This specific pyroptotic pathway actively remodels the tumor microenvironment by driving the CXCL2-dependent recruitment of myeloid-derived suppressor cells (MDSCs), thereby creating an immunosuppressive niche that facilitates disease progression.^426^ GSDMC also exerts antitumor effects via caspase-8-mediated pyroptosis. Under hypoxic conditions, p-STAT3 physically interacts with PD-L1 to facilitate its nuclear translocation, thereby enhancing the transcription of the *GSDMC* gene. Once expressed, GSDMC is cleaved by caspase-8 following TNFα stimulation.^150^ Moreover, the metabolite α-ketoglutarate (α-KG) elevates ROS levels to induce DR6 endocytosis, leading to caspase-8/GSDMC-mediated pyroptosis and the suppression of metastasis in vivo.^151^ GSDMD has been identified as a suppressor of gastric cancer (GC) cell proliferation.^427^ Moreover, recent findings have demonstrated that the transcriptional regulator hypermethylated in cancer 1 (HIC1) directly upregulates GSDMD expression, promoting pyroptosis, which significantly enhances the infiltration of cytotoxic CD8^+^ T cells. Although this axis is frequently compromised by Jab1-mediated proteasomal degradation of HIC1, combining HIC1 overexpression with anti-PD-L1 therapy exerts a profound synergistic antitumor impact.^428^ In colon cancer, the conventional lipid-lowering drug simvastatin exhibits potent antitumor activity by driving the production of ROS, which subsequently activates the canonical caspase-1/GSDMD pathway, inducing robust tumor cell pyroptosis both in vitro and in vivo.^429^ High levels of GSDME serve as a predictor of poor prognosis in both hepatocellular carcinoma (HCC) and clear cell renal cell carcinoma (ccRCC).^1^ A recent study identified that arachidonic acid (AA) triggers irreversible mitochondrial permeability transition, which subsequently activates the caspase-3/GSDME pathway to induce pan-cancer pyroptosis, thereby remodeling immunosuppressive tumor microenvironments, boosting cytotoxic T-cell infiltration, and synergizing with anti-PD-1 therapy in immunocompetent models.^430^ However, in the context of treatment, caspase-3-mediated cleavage of GSDME converts chemotherapy-induced apoptosis into pyroptosis. This process releases inflammatory mediators (IL-1β, IL-18, HMGB1, ATP) and tumor-associated antigens, which promote dendritic cell maturation, enhance antigen presentation, and drive the activation of CD8^+^ T cells and NK cells.^24,155,431–433^ In conclusion, pyroptosis acts as a double-edged sword in tumorigenesis. While it potentiates antitumor defenses through immunogenic cell death, it can also facilitate immunosuppressive TME remodeling and therapeutic resistance. The ultimate impact is dictated by specific molecular contexts, induction pathways, and microenvironmental cues. Thus, pyroptosis serves as a critical mediator of tumor-immune interactions and represents a promising therapeutic target that demands context-specific modulation.

Altogether, the evidence presented in this section underscores the multifaceted nature of GSDM-mediated pyroptosis across diverse pathologies. The function of this pathway is strictly context dependent. It is neither inherently beneficial nor harmful but rather dictated by cell type, disease etiology, and progression stage. Consequently, future translational medicine must focus on developing precision therapies that can selectively modulate specific GSDM pathways within defined microenvironments.

## Non-pyroptotic functions of gasdermins in health and disease

While GSDMs have been firmly linked to cell death, emerging research reveals far more intricate and diverse functions. Notably, pore formation does not always lead to membrane rupture. In this section, we will offer an overview of the remarkable functions of GSDMs, driven by their context-dependent cleavage into distinct fragments that regulate processes as diverse as immune activity, bone resorption, mucosal barrier integrity, and food tolerance.

### Gasdermins in killing bacteria

**Fig. 5:**
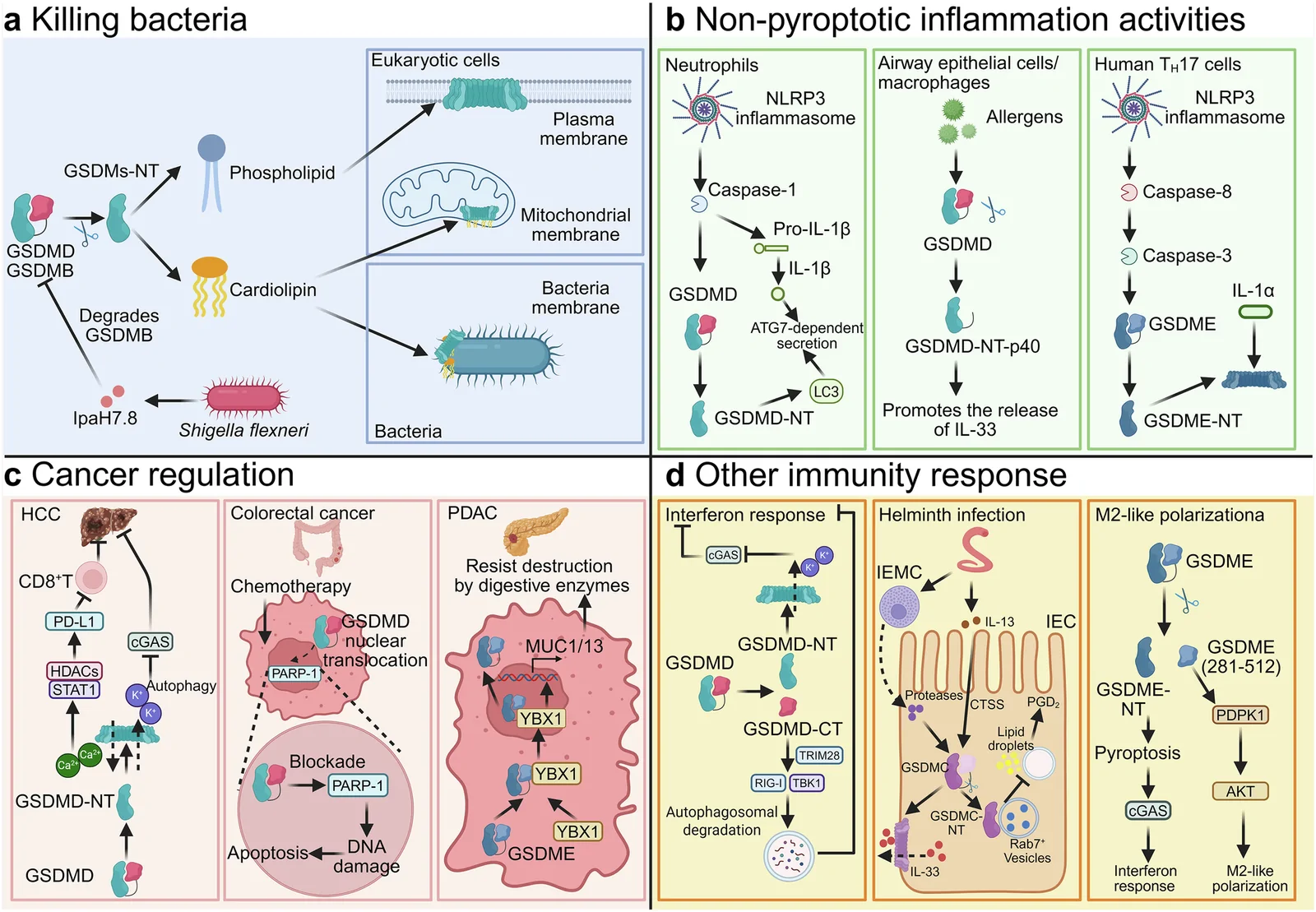
GSDMs in killing bacteria, non-pyroptotic inflammation activities, cancer regulation and other immune responses. **a** GSDMD-NT and GSDMB-NT fragments, which have a strong affinity for cardiolipin in bacterial membranes, kill intracellular bacteria by perforating their membranes. However, as a counter-defense, *Shigella* uses the protein IpaH7.8 to specifically degrade GSDMB, protecting itself from this mechanism. **b** In neutrophils, GSDMD facilitates IL-1β release by targeting LC3^+^ autophagosomes without inducing pyroptosis. In airway epithelial cells and macrophages, allergenic proteases cleave GSDMD to produce a novel fragment, GSDMD-NT-p40 (p35 in humans), promoting the release of IL-33 without causing cell death. In human T cells, the NLRP3/caspase-8/caspase-3/GSDME pathway is essential for GSDME-NT to form pores that mediate the unconventional release of IL-1α. **c** In hepatocellular carcinoma (HCC), the formation of GSDMD-NT pores helps tumors survive by exporting potassium ions to promote autophagy, which dampens the cGAS pathway, and by importing calcium ions, which activates a signaling cascade that increases PD-L1 expression to help the tumor evade the immune system. In colorectal cancer, chemotherapy drugs stimulate the nuclear translocation of GSDMD, which promotes apoptosis by blocking PARP-1 to inhibit DNA damage repair. In human pancreatic ductal adenocarcinoma (PDAC) cells, GSDME helps the tumor resist destruction by digestive enzymes by promoting the expression of mucin 1 and mucin 13, which form a protective barrier. **d** GSDMD suppresses the cGAS-driven type-I interferon response to cytosolic DNA by both its pore-forming domain, which depletes intracellular potassium, and its C-terminal domain, which targets key signaling proteins for autophagosomal degradation. Under type 2 immune induction, GSDMC is cleaved by cathepsin S (CTSS), and the resulting NT fragment targets late endosomes to block the transport of lipid droplets for lysosomal degradation, thereby restricting the production of immunosuppressive lipids and promoting an amplified anti-helminth type 2 immune response. In macrophages, a non-NT fragment of GSDME combines with PDPK1 to activate the PI3K-AKT pathway, which drives M2-like polarization. This figure is created in BioRender

Emerging evidence has revealed a more direct role for GSDMs in fighting infection. GSDM-NT fragments possess intrinsic bactericidal activity, enabling them to kill bacteria directly by forming pores in their membranes. This molecular mechanism relies on the lipid-binding specificity of the GSDM-NT domain, which enables it to distinguish between host and microbial membranes. While GSDMD-NT targets the host cell’s plasma membrane by binding to inner-leaflet phospholipids such as PIPs, it also exhibits a strong affinity for cardiolipin (CL).^180^ This is a key distinction because cardiolipin is found almost exclusively in the inner mitochondrial membrane of eukaryotic cells, but it is an abundant component of the inner and outer membranes of many bacteria. This differential lipid distribution allows GSDMD-NT to selectively target and perforate the membranes of intracellular bacteria (Fig. 5a).^20,130^ Furthermore, upon pyroptosis, extracellular GSDMD-NT has the potential to act as an antimicrobial peptide, although its half-life and efficacy in the complex extracellular environment remain to be fully defined. This direct antimicrobial function is not limited to GSDMD. GSDMB exhibits potent, direct bactericidal activity by recognizing phospholipids on gram-negative bacterial membranes. However, this is counteracted by the *Shigella* protein IpaH7.8, a ubiquitin ligase that specifically targets GSDMB for proteasomal degradation, thus protecting the bacterium from this killing mechanism (Fig. 5a).^146^ The recent discovery of functional GSDM homologs in bacteria, which are activated by dedicated proteases to defend against bacteriophage infection, suggests that membrane perforation as a defense strategy is a deeply conserved mechanism shared between prokaryotes and animals.^172^

### Gasdermins in non-pyroptotic inflammatory activities

Gasdermins, particularly GSDMD, are increasingly recognized for their crucial roles in inflammation-related immune activities.

The IL-1β cytokine is primarily generated via the proteolytic cleavage of pro-IL-1β by caspase-1, with its subsequent secretion mediated by the GSDMD-NT pore. In neutrophils, GSDMD facilitates IL-1β release by targeting LC3^+^ autophagosomes, notably without inducing pyroptosis (Fig. 5b).^106,131^ Furthermore, recent electron microscopic and functional assessments of the GSDMD-NT pore have elucidated a mechanism that specifically prevents pro-IL-1β release. The GSDMD-NT pore mediates the selective efflux of mature IL-1β through electrostatic filtration, thereby hindering pro-IL-1β flux.^197^ GSDM pores facilitate the secretion of inflammatory mediators such as IL-1α and IL-33 from living cells.^134,434,435^ In airway epithelial cells and macrophages, allergenic proteases cleave GSDMD to produce a novel fragment, GSDMD-NT-p40 (p35 in humans).^134^ This fragment promotes the release of IL-33 without causing cell death (Fig. 5b). In human T cells, the NLRP3/caspase-8/caspase-3/GSDME axis is crucial for GSDME-NT pores to mediate the unconventional release of IL-1α (Fig. 5b).^435^ Additionally, GSDMs also drive the unconventional release of other inflammatory molecules, such as ATP and HMGB1.^436^

A state termed hyperactivation has been described, in which cells, particularly professional phagocytes such as macrophages, dendritic cells (DCs), and neutrophils, can sustain GSDM pore formation in their plasma membrane while remaining viable and functionally active.^124,177^ Endothelial cells are also found to be hyperactivated when protein kinase R (PKR) is activated, leading to the release of HMGB1 and IL-1β.^437^ This sublytic activity transforms the GSDM pore from an instrument of death into a regulated channel for the unconventional release of inflammatory signals, allowing the cell to sound a persistent ‘alarm bell’ without sacrificing itself.

### Gasdermins in pyroptosis-independent cancer regulation

While the execution of pyroptosis significantly impacts tumorigenesis (as discussed in the previous sections), in this part, we will focus on research exploring the pyroptosis-independent functions of GSDMs in cancer. In glioma, GSDMA expression correlates with the infiltration of CD8^+^ T cells and regulatory T cells (Tregs), serving as a predictor of poor prognosis.^438^ Additionally, in esophageal squamous cell carcinoma (ESCC), FAM111B has been shown to directly bind GSDMA, thereby promoting tumor progression.^439^ In bladder cancer cells, GSDMB interacts with STAT3 to increase STAT3 phosphorylation and reprogram glucose metabolism, promoting tumor growth.^223^ Conversely, GSDMB has been demonstrated to exert antitumor effects. GSDMB interacts with insulin-like growth factor 2 mRNA-binding protein 1 (IGF2BP1), which directly binds to and recognizes the 3’-UTR of *DUSP6* mRNA. This interaction enhances *DUSP6* translation, subsequently inhibiting ERK phosphorylation to promote cell death and restrain proliferation.^440^ In a mouse model of obesity-induced hepatocellular carcinoma (HCC), IL-33 is released through GSDMD pores from senescent hepatic stellate cells (HSCs), promoting HCC development by activating ST2-positive regulatory T cells within the liver TME. The accumulation of GSDMD-NT has also been detected in HSCs from patients with non-alcoholic steatohepatitis (NASH)-associated HCC, suggesting that a similar pathogenic mechanism contributes to specific subtypes of human HCC.^434^ It has also been reported that GSDMD critically regulates the tumor immune microenvironment through pore-mediated ion flux. Although acting via distinct ionic mechanisms, GSDMD activity functionally converges to suppress antitumor immunity. On the one hand, GSDMD-mediated calcium influx activates a calcium-dependent histone deacetylase (HDAC)/STAT1 axis, leading to the transcriptional transactivation of the PD-L1 gene. Concurrently, GSDMD-driven potassium efflux promotes autophagy, which dampens cGAS activation and type I interferon production (Fig. 5c).^441^ This immunosuppressive function extends to antigen-presenting cells (APCs). Recent work has demonstrated that GSDMD restricts cGAS-dependent signaling in APCs, thereby limiting the expression of interferon-stimulated genes (ISGs) and subsequent CD8^+^ T-cell activation.^442^ Thus, in both tumor cells and APCs, GSDMD exploits ionic homeostasis and cGAS signaling to restrict antitumor immunity. In CD4^+^ T cells, T-cell receptor stimulation activates caspase-8 to cleave GSDMD, and the GSDMD pores facilitate a controlled Ca^2+^ influx that is essential for IL-2 induction, which enables CD4^+^ T cells to provide critical help to CD8^+^ T cells, thereby sustaining antitumor immunity. Notably, this nonpyroptotic activation of GSDMD in human CD4^+^ T cells correlates with favorable prognoses and improved responses to anti-PD-1 immunotherapy in colonic and pancreatic cancers.^443^ GSDMD also influences cancer cell survival through non-canonical subcellular localization and signaling. In colorectal cancer, chemotherapy stimulates the nuclear translocation of GSDMD. Once in the nucleus, GSDMD interacts with PARP-1 to block its DNA repair function, establishing a hypoxia/chemotherapy-GSDMD-PARP-1 axis that drives apoptosis (Fig. 5c).^255^ Conversely, in non-small cell lung cancer (NSCLC), GSDMD depletion activates caspase-3 and PARP cleavage via the intrinsic mitochondrial pathway, suggesting that GSDMD may normally suppress mitochondrial apoptosis in this context.

Other GSDM proteins have also been linked to various cancers. GSDMA interacts with FAM111B and regulates tumorigenesis and chemosensitivity in esophageal squamous cell carcinoma (ESCA).^439^ In human pancreatic ductal adenocarcinoma (PDAC) cells, GSDME is utilized to resist destruction by digestive enzymes. GSDME achieves this by helping tumor cells express mucin 1 and mucin 13, which are proteins that form a protective barrier against enzymes such as chymotrypsin, allowing tumor cells to survive in the harsh digestive environment (Fig. 5c).^444^

### Gasdermins in other immune pathways

GSDMD has also been shown to influence other immune pathways, particularly the type-I interferon response. In one context, GSDMD activated by the AIM2 inflammasome was found to suppress the cGAS-driven type-I interferon response to cytosolic DNA. It achieves this by using its membrane pores to deplete intracellular potassium, an action that is both necessary and sufficient to inhibit the IFN-β response.^35^ In a different mechanism, the C-terminal domain of GSDMD (GSDMD-CT) is reported to inhibit the type-I interferon response by targeting key signaling proteins, RIG-I and TBK1, for autophagosomal degradation (Fig. 5d).^445^ Additionally, GSDMD could dampen coronavirus infection by promoting the nonclassical release of antiviral IFN-β, which in turn enhances interferon-stimulated gene (ISG) responses.^446^ Recent studies also indicate that GSDMD has a pivotal role in promoting lung neutrophil responses, thereby amplifying inflammation and pathogenesis in influenza virus infections.^329^

In the early stages of *Leishmania* infection, GSDMD becomes active in macrophages, leading to transient cell permeabilization and potassium efflux. This process activates the NLRP3 inflammasome without triggering pyroptosis. During this time, GSDMD is also cleaved into a non-canonical 25 kDa fragment. Supporting its critical role, macrophages and mice lacking GSDMD exhibit reduced NLRP3 inflammasome activation and are highly susceptible to several *Leishmania* species.^133^

Upon exposure to allergens, a newly formed murine amino-terminal p40 fragment of GSDMD mediates the secretion of IL-33 from the cytosol in epithelial cells. This process, which involves pore formation in the cell membrane, is notable because the generation of the p40 fragment is independent of the inflammatory caspase-1 and caspase-11 pathways. The release of IL-33 can be effectively prevented by either blocking stress granule assembly or by abolishing p40 production through a mutation of the ELRQQ residues at positions 309–313.^134^

Studies have also reported on the roles of other GSDM proteins in other immune activities. A study revealed a correlation between GSDMB expression and genes associated with IFN signaling, MHC class I antigen presentation, and other immune system pathways.^346^ During helminth infections in the gut, αEβ7 integrin-positive intraepithelial mast cells (IEMCs) engage in extensive crosstalk with neighboring intestinal epithelial cells (IECs). Proteases released by IEMCs cleave GSDMC proteins within IECs, a process that releases IL-33. Notably, mast cell deficiency abolishes this GSDMC-mediated immune cascade.^154^ Another study revealed a more intricate mechanism. Under type 2 immune induction, GSDMC is directly cleaved by cathepsin S (CTSS). The resulting N-terminal fragment targets Rab7⁺ late endosomes, disrupting their membrane integrity. This disruption blocks the transport of lipid droplets for lysosomal degradation, which in turn restricts the production of the immunosuppressive lipid PGD₂. This pathway promotes goblet and villous enterocyte hyperplasia and an IL-13⁺ ILC2/Th2 response, which collectively amplifies anti-helminth type 2 immunity (Fig. 5d).^447^ GSDME-dependent pyroptosis promotes cell death and the release of mitochondrial DNA, which activates cGAS-STING signaling and triggers a robust interferon response and myocardial inflammation.^448^ GSDME also influences macrophage polarization. A non-N-terminal fragment (281–512 aa) of GSDME (mouse) combines with PDPK1, activating the PI3K-AKT pathway and facilitating M2-like polarization within macrophages (Fig. 5d). This mechanism is clinically relevant, as studies in hepatocellular carcinoma (HCC) show that suppressing GSDME expression in non-tumor cells decreases the proportion of M2-like macrophages in the tumor microenvironment while concurrently enhancing the cytotoxicity of CD8⁺ T cells.^449^

### Gasdermins in tissue repair and stability

A series of groundbreaking studies revealed that GSDMs, especially GSDMD, mediate the release of pro-regenerative and inflammation-resolving molecules. The same pore that releases the inflammatory cytokines also serves as a conduit for “healing” signals, challenging the traditional dogma that GSDMs are purely agents of inflammation and tissue damage.

**Fig. 6:**
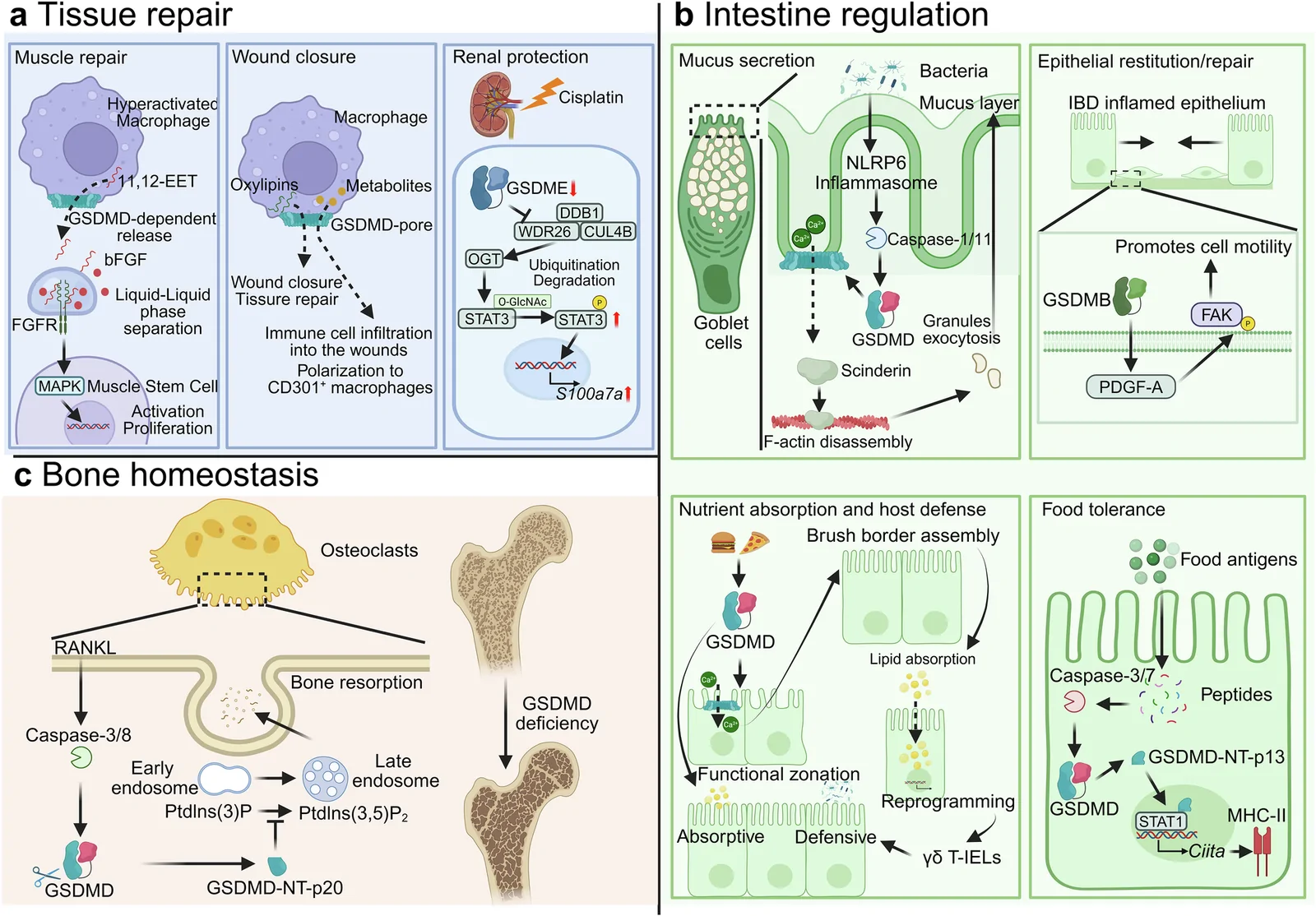
GSDMs in tissue repair, intestine regulation and bone homeostasis. **a** 11,12-epoxyeicosatrienoic acid (11,12-EET) accelerates muscle regeneration by enhancing fibroblast growth factor signaling and boosting muscle stem cell activity. Inducing pyroptosis in macrophages results in a beneficial secretome rich in oxylipins and metabolites, which is released through GSDMD pores and accelerates wound healing by promoting cell migration and proliferation. GSDME deficiency in the kidney destabilizes the protein OGT and leads to increased macrophage infiltration, thereby aggravating cisplatin-induced injury. **b** In goblet cells, GSDMD is cleaved to form the GSDMD-NT fragment, which creates sublytic pores that facilitate calcium influx to activate Scinderin, a protein that disassembles the F-actin network to enable the release of mucin for a protective mucus layer. GSDMB is crucial for repairing the barrier by regulating the PDGF-A-FAK signaling axis to enhance epithelial cell proliferation and migration for wound closure. Epithelial GSDMD in the small intestine facilitates nutrient absorption and brush border assembly, a process that supports intraepithelial γδ T lymphocytes and balances nutrient uptake with host defense. In intestinal epithelial cells, a GSDMD fragment cleaved by caspase-3/7 translocates to the nucleus to induce MHCII transcription, a process that is crucial for the induction of Tr1 cells and the maintenance of food tolerance. **c** During osteoclast development, the GSDMD-NT-p20 fragment is produced in a RIPK1/caspase-8/caspase-3-dependent manner and functions to protect against excessive bone loss by inhibiting the maturation of early endosomes into lysosomes, which limits the secretion of bone-degrading enzymes. This figure is created in BioRender

Recent work has identified a non-canonical, metabolite-secreting function for GSDMD in hyperactivated macrophages that is essential for tissue repair. 11,12-epoxyeicosatrienoic acid (11,12-EET) has been identified as a key pro-regenerative factor secreted in a GSDMD-dependent manner. This metabolite accelerates muscle regeneration by enhancing fibroblast growth factor signaling and modulating its liquid‒liquid phase separation, which in turn boosts muscle stem cell activity (Fig. 6a).^450^ In a separate but related discovery, another study found that inducing pyroptosis in macrophages without the release of pro-inflammatory IL-1β or IL-1α results in a beneficial secretome rich in oxylipins and metabolites. This secretome promotes cell migration and proliferation, accelerating wound healing both in vitro and in vivo. It was determined that a key component, prostaglandin E2 (PGE2), is synthesized late during pyroptosis in a caspase-1- and cyclooxygenase-2-dependent manner and is released through GSDMD pores (Fig. 6a).^451^ Additionally, GSDME also demonstrates a protective, non-pyroptotic function. Research shows that a loss of GSDME in kidney tubules aggravates cisplatin-induced injury. Mechanistically, GSDME absence destabilizes the protein OGT, disrupting STAT3 modification and causing an increase in S100A7A. This excess S100A7A promotes macrophage infiltration damage, highlighting a novel GSDME-dependent pathway that protects the kidney from inflammation (Fig. 6a).^452^

### Gasdermins in the intestine

The intestinal epithelium is a critical barrier separating the host from the gut microbes. Maintaining its integrity is vital for health, and surprisingly, GSDMs play distinct, non-pyroptotic roles in this process. The primary chemical barrier in the colon is a thick layer of mucus, which is secreted by specialized epithelial cells known as goblet cells.^453^ Recent research has identified GSDMD as a key component of the cellular machinery for mucus secretion. In goblet cells, various stimuli trigger caspase activation and subsequent GSDMD cleavage. The resulting GSDMD-NT fragment forms a limited number of sublytic pores in the apical plasma membrane. These pores facilitate a rapid influx of calcium ions, which activates Scinderin, a protein that disassembles the cortical F-actin network. This disassembly is a critical prerequisite for granule exocytosis, enabling the release of mucin to form the protective mucus layer (Fig. 6b).^454^ While GSDMD helps build the barrier, GSDMB is crucial for repairing it. GSDMB expression is significantly upregulated in the inflamed epithelium of patients with IBD. Contrary to expectations for gasdermin, this increase does not cause more pyroptosis. Instead, GSDMB enhances epithelial cell proliferation and migration, which are essential for wound closure. Mechanistically, GSDMB regulates the PDGF-A-FAK signaling axis, which promotes cell motility by controlling focal adhesion turnover. By modulating this pathway, GSDMB ensures that epithelial cells can dynamically remodel their adhesions and migrate effectively to repair the barrier (Fig. 6b).^37^

A study found that a 13-kD N-terminal fragment GSDMD, cleaved by caspase-3/7 in intestinal epithelial cells, translocates to the nucleus. This fragment then induces the transcription of MHCII molecules, which in turn leads to the induction of Tr1 cells in the small intestine. This GSDMD-dependent pathway is crucial for maintaining food tolerance, demonstrating that differential GSDMD cleavage acts as a regulatory hub to balance immunity and tolerance in the gut (Fig. 6b).^50^ Recent research has also revealed a crucial non-pyroptotic function for epithelial GSDMD in the small intestine. GSDMD facilitates nutrient absorption and assembly of the brush border, a process that supports intraepithelial γδ T lymphocytes (γδ T-IELs). Consequently, this GSDMD-mediated circuit is essential for balancing nutrient uptake with host defense in response to dietary changes, and its impairment leads to severe gut inflammation (Fig. 6b).^455^

### Other functions of gasdermins

A recent study revealed a function for GSDMD in bone homeostasis. During osteoclast development, the protein is cleaved by caspase-3 and caspase-8 in a RIPK1-dependent manner to produce a GSDMD-NT-p20 fragment. Instead of causing cell death, GSDMD-NT-p20 acts as a brake on bone resorption. It inhibits the maturation of early endosomes into lysosomes by limiting phosphoinositide conversion and binding to PI(3)P, which effectively limits the secretion of bone-degrading enzymes, thereby protecting against excessive bone loss (Fig. 6c).^51^

GSDME is associated with progressive hearing loss through a toxic gain-of-function mechanism; mutations cause a constitutively active form of the protein that forms pores in cell and mitochondrial membranes, leading to the progressive death of hair cells.^456^ DFNB59 (Pejvakin) is a crucial peroxisome-associated protein that protects auditory cells from noise-induced damage. It functions as a redox-activated receptor, sensing oxidative stress and initiating pexophagy to degrade damaged peroxisomes. A loss of this function leads to the accumulation of damaged peroxisomes and increased oxidative stress, making auditory cells exceptionally vulnerable to sound and ultimately causing hearing loss.^168,169^

The functions of GSDMs are critically determined by which proteolytic fragment is generated and where it subsequently translocates within the cell (Table 4). The N-terminal fragment of GSDMD, for instance, typically moves to the plasma membrane to form pores and execute pyroptosis. However, this same fragment can be redirected to mitochondrial membranes to trigger necroptosis, while distinct, smaller GSDMD fragments can travel to the nucleus to either promote apoptosis by blocking DNA repair or maintain food tolerance. This principle of location-dependent function extends to other family members as well. The GSDME-NT fragment can target either the plasma membrane to cause pyroptosis or the mitochondria to amplify apoptosis, while the GSDMC-NT fragment targets the membranes of late endosomes to modulate immunity. Therefore, the specific cleavage event and the resulting fragment’s final destination are the critical determinants that switch GSDM functions between cell death, immune signaling, and tissue homeostasis.

**Table 4:** GSDMs fragments and their non-pyroptotic functions

Gasdermin	Fragment/form	Location	Function	Refs
GSDMA	N-Terminal	Plasma Membrane	Induces pyroptosis when cleaved by the bacterial toxin SpeB	^16,17^
GSDMB	N-Terminal	Plasma Membrane	Triggers pyroptosis when cleaved by granzyme A from immune cells	^18,25^
		Bacterial Membrane	Kills bacteria directly by forming pores in their membranes	^146^
	Full-Length	Intestinal Epithelium	Promotes epithelial cell migration and wound repair, independent of pyroptosis	^37^
GSDMC	N-Terminal	Plasma Membrane	Induces pyroptosis when cleaved by Caspase-8	^150^
		Late Endosomes	Targets late endosomes to amplify the immune response against helminths	^447^
GSDMD	N-Terminal	Plasma Membrane	The primary executioner of pyroptosis by forming pores	^13,14^
			Mediates the non-lethal release of inflammatory molecules	^177^
		Mitochondrial Membranes	Can amplify apoptosis by permeabilizing mitochondria	^36,180^
			Can trigger necroptosis when redirected there by high oxidative stress	^262^
		Bacterial Membranes	Directly kills bacteria like *E. coli* and *S. aureus* by forming pores	^20,130^
		Intestinal Goblet Cell Apical Membrane	Forms temporary pores to allow calcium influx, which is necessary for mucus secretion	^454^
	N-Terminal p20	Osteoclast Endosomes	Inhibits the maturation of lysosomes, thereby reducing bone resorption and maintaining bone mass	^51^
	N-Terminal p40	Lung epithelial cells	Forms pores, releasing IL-33 and activating type Ⅱ immunity	^134^
	N-Terminal p25	Macrophages’ membrane (*Leishmania* infection)	Permeabilizes cells to cause K^+^ efflux and activate the NLRP3 inflammasome, but does not induce cell death	^133^
	N-Terminal p13	Nucleus (Intestinal Epithelial Cells)	Induces MHCII transcription to help maintain food tolerance; does not cause pyroptosis	^50^
	C-Terminal	Cytosol/Autophagosomes	Inhibits the type-I interferon response by targeting signaling proteins for degradation	^445^
	Full-Length	Nucleus (Colorectal Cancer Cells)	Promotes apoptosis by blocking the DNA repair protein PARP-1, making cancer cells more sensitive to chemotherapy	^255^
		Intestinal Epithelium	Facilitates nutrient absorption and supports local immune cells (γδ T-IELs)	^455^
GSDME (DFNA5)	N-Terminal (NT)	Plasma Membrane	Switches apoptosis to pyroptosis when cleaved by Caspase-3	^23,24^
		Mitochondrial Membranes	Permeabilizes mitochondria to release cytochrome C, amplifying the apoptotic signal	^36^
	Non-N-Terminal (aa 281–512)	Cytosol (Macrophages)	Associates with PDPK1 to activate the PI3K-AKT pathway, promoting M2-like macrophage polarization	^449^
	Full-Length (Mutated)	Plasma & Mitochondrial Membranes (Auditory Hair Cells)	Gain-of-function mutations cause a constitutively active protein that forms pores, leading to cell death and progressive hearing loss	^456^
	Full-Length	Kidney Tubules	Protects the kidney from inflammation and cisplatin-induced injury	^452^
DFNB59 (Pejvakin)	Full-Length	Peroxisomes (Auditory Hair Cells)	Acts as a sensor for oxidative stress, protecting auditory cells by initiating the removal of damaged peroxisomes (pexophagy)	^168,169^

## Emerging targets and therapeutic interventions involving gasdermins

Uncovering the dual pyroptotic and non-pyroptotic functions of GSDMs highlights their potential as versatile therapeutic targets. Approaches that employ specific small-molecule modulators or novel material-based delivery systems to exert precise control are becoming increasingly central to the future of therapeutic development.

### GSDMD inhibitors and activators

**Fig. 7:**
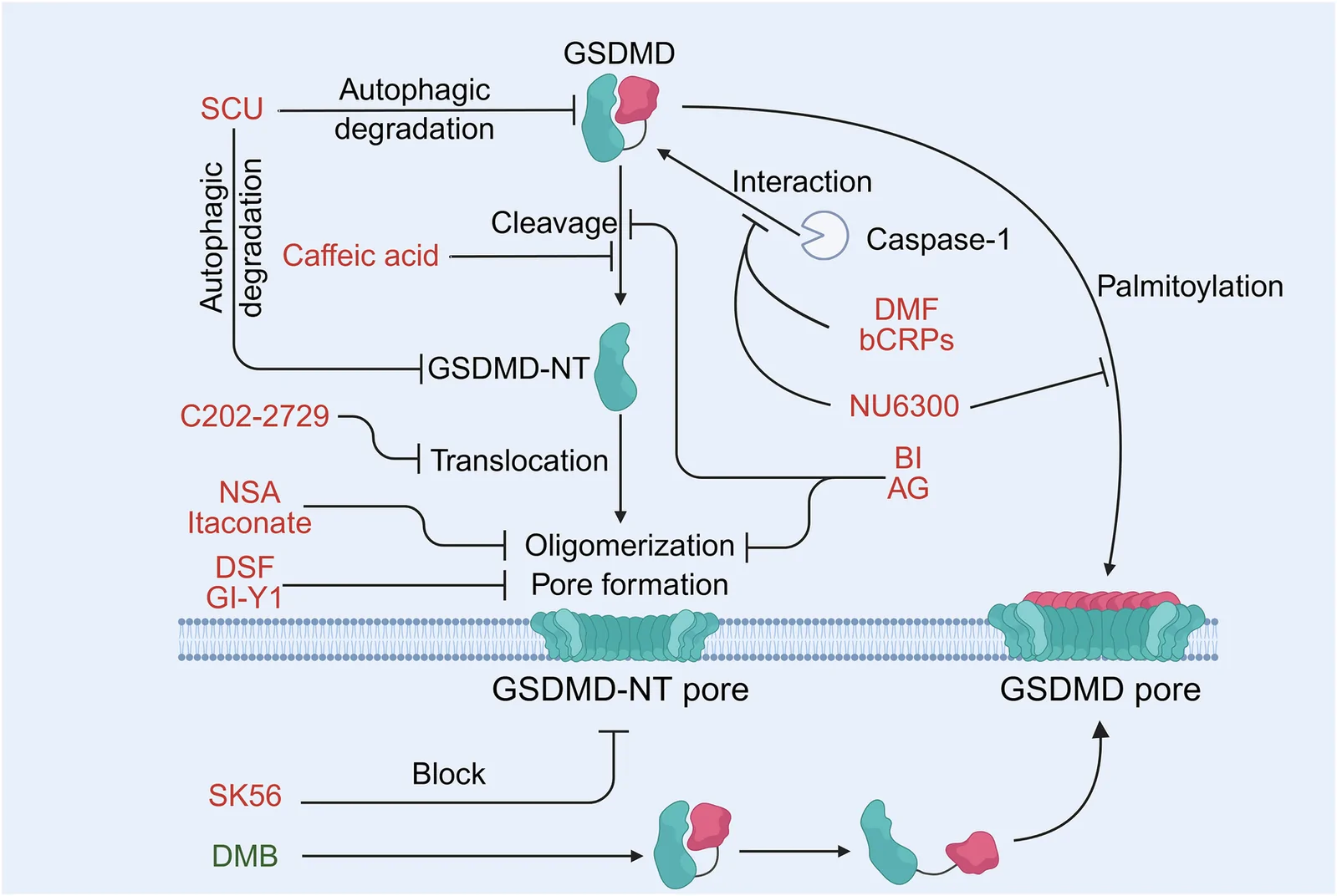
GSDMD-specific inhibitors and activators. GSDMD typically undergoes caspase-1 cleavage to generate an NT fragment, which translocates to the plasma membrane to oligomerize and form pores. Pharmacological agents target specific stages of this pathway. Dimethyl fumarate (DMF) and bicyclic cysteine-rich peptides (bCRPs) prevent GSDMD processing by blocking its interaction with caspase-1. Caffeic acid, NU6300, baohuoside I (BI), and andrographolide (AG) inhibit caspase-1-mediated cleavage. Notably, NU6300 also inhibits GSDMD palmitoylation, while BI and AG dually inhibit GSDMD-NT oligomerization. Scutellarin (SCU) promotes the autophagic degradation of both full-length GSDMD and GSDMD-NT. C202-2729 blocks membrane translocation. Necrosulfonamide (NSA) and disulfiram (DSF) target critical cysteine residues (Cys191/192) to prevent oligomerization, while itaconate targets Cys77 to inhibit pore formation. GI-Y1 disrupts the GSDMD-NT/lipid interface, while SK56 directly obstructs the pore to prevent cytokine release. In contrast, the compound DMB acts as an activator, triggering GSDMD pore formation independent of proteolytic cleavage. This figure is created in BioRender

Necrosulfonamide (NSA) is the first specific GSDMD inhibitor to be identified.^457^ Originally characterized as an inhibitor of MLKL-driven necroptosis by binding to Cys86, NSA was subsequently found to directly bind Cys191 (human) or Cys192 (mouse) of GSDMD. This interaction effectively blocks GSDMD oligomerization without affecting its proteolytic cleavage (Fig. 7).^457,458^ However, given that Cys191/192 are critical sites for GSDMD palmitoylation, it warrants investigation whether NSA exerts its effects by interfering with this post-translational modification. Furthermore, subsequent studies indicate that NSA also suppresses LPS-induced gene transcription and caspase-1 activation, suggesting that its activity may not be exclusively limited to GSDMD. Despite these mechanistic nuances, NSA has demonstrated significant therapeutic efficacy, alleviating disease severity in multiple models, including DSS-induced colitis, DHT-induced hair growth inhibition, and sepsis-associated encephalopathy.^459–461^

Disulfiram (DSF) is another well-established, specific inhibitor of GSDMD. Similar to NSA, DSF targets C191/192 of GSDMD. Original studies demonstrated that DSF covalently modifies these residues to block pore formation without affecting GSDMD cleavage or earlier events in the inflammasome pathway (Fig. 7).^462^ However, recent evidence challenges this specificity, as DSF has been found to inhibit TLR4 signaling by covalently modifying Cys133 of MD-2, a key conserved residue essential for TLR4 sensing and signaling.^463^ DSF is an FDA-approved drug, and its therapeutic function has been reported in various disease models, such as LPS-induced sepsis, nonalcoholic steatohepatitis and atherosclerosis.^464–466^ NU6300 has also been found to covalently target Cys191 of human GSDMD to block caspase-1-mediated cleavage. Additionally, it inhibits the interaction of GSDMD with ZDHHC5, thereby suppressing the palmitoylation required for efficient pore formation (Fig. 7).^467^

Dimethyl fumarate (DMF) has been found to react with GSDMD at critical cysteine residues to form S-(2-succinyl)-cysteine. This succination event prevents the interaction between GSDMD and caspases, thereby limiting GSDMD processing, oligomerization, and the capacity to induce cell death (Fig. 7). In vivo, the administration of DMF protects against LPS-induced shock and alleviates disease severity in murine models of familial Mediterranean fever and experimental autoimmune encephalitis by specifically targeting GSDMD. Moreover, DMF also succinates GSDME at Cys45 and other sites to prevent GSDME cleavage and GSDME-dependent pyroptosis.^244^ DMF also exerts anti-inflammatory effects by succinating the catalytic cysteine of GAPDH. This inactivation downregulates aerobic glycolysis in activated myeloid and lymphoid cells, thereby dampening their inflammatory response.^468^ The potential therapeutic efficacy of DMF in Alzheimer’s disease has also been investigated.^469^

Scutellarin (SCU) has recently been identified as a pyroptosis inhibitor that promotes the degradation of both GSDMD-NT and full-length GSDMD. Notably, SCU exhibits a higher affinity for degrading the active GSDMD-NT fragment than the full-length GSDMD. Mechanistically, SCU recruits TRIM21 to catalyze the K33-linked ubiquitination of GSDMD at residue Lys51. This modification serves as a specific recognition signal for the cargo receptor SQSTM1/p62, thereby targeting GSDMD for autophagic degradation (Fig. 7).^237^

Itaconate binds to GSDMD at Cys77, which is critical for oligomerization. This interaction disrupts pore formation and may also interfere with caspase-GSDMD binding, thereby inhibiting pyroptosis (Fig. 7).^245^ Caffeic acid has been reported to prevent GSDMD cleavage to inhibit pyroptosis.^470^ Emestrin-type epidithiodiketopiperazines (ETPs) have been reported to activate caspase-3/7, which in turn cleaves human GSDMD at Asp87 to generate a p10 fragment, thereby inhibiting GSDMD-NT generation.^471^ Liu et al. developed a novel high-throughput hGLuc-mGSDMD-PCA screening system utilizing coelenterazine as a substrate to sensitively monitor GSDMD-NT oligomerization via a luciferase signal. This screen identifies 17 compounds capable of suppressing both GSDMD cleavage and GSDMD-NT oligomerization, with baohuoside I (BI) and andrographolide (AG) demonstrating the highest potency (Fig. 7).^472^ Targeting the terminal steps, C202-2729 prevents the translocation of GSDMD-NT to the plasma membrane, while GI-Y1 disrupts the binding interface between GSDMD-NT and the membrane (Fig. 7).^473,474^ A recent study utilizing artificial intelligence screening identified SK56, a peptide that effectively inhibits pyroptosis and cytokine release by directly obstructing GSDMD-NT pores. Crucially, SK56 does not affect the upstream cleavage of GSDMD but blocks pore integrity. This inhibition suppresses cytokine release from pyroptotic macrophages, mitigates dendritic cell activation triggered by pyroptotic cell membranes, and prevents widespread cell death in a human alveolar organoid-macrophage co-culture model. Mechanistically, SK56 has been shown to block GSDMD-NT pores on lipid bilayer nanoparticles and enter pyroptotic macrophages to protect against mitochondrial damage (Fig. 7).^475^ Recently, mRNA-displayed libraries of bicyclic cysteine-rich peptides (bCRPs) have been developed. Researchers utilize these libraries to engineer potent peptide ligands for full-length GSDMD through a two-stage discovery process. Initial hit compounds are identified de novo via GSDMD affinity selection using the Random Nonstandard Peptides Integrated Discovery system, followed by optimization through mRNA display-based saturation mutagenesis. The resulting bCRPs bind to full-length GSDMD with high affinity and prevent GSDMD from cleavage by caspases (Fig. 7).^476^

Beyond the use of inhibitors, activating GSDMD represents a promising therapeutic strategy, particularly in cancer. The compound 6,7-dichloro-2-methylsulfonyl-3-N-tert-butylaminoquinoxaline (DMB) was identified as a direct and selective GSDMD agonist. Mechanistically, DMB triggers GSDMD pore formation and pyroptosis without requiring proteolytic cleavage (Fig. 7). In vivo, DMB-mediated pulsed, low-level pyroptosis inhibits tumor progression without compromising the viability of GSDMD-expressing immune populations. The observed antitumor effect is strictly immune-mediated, as evidenced by the abrogation of protection in lymphocyte-deficient mice. Furthermore, vaccination with DMB-treated cancer cells conferred robust protection against secondary tumor challenge, confirming the induction of immunogenic cell death. DMB treatment also demonstrated synergy with anti-PD-1 therapy while maintaining a favorable safety profile.^477^ Other drugs that activate pyroptosis indirectly also demonstrate therapeutic potential. For instance, paroxetine hydrochloride inhibits the reuptake of 5-hydroxytryptamine (5-HT), a driver of melanoma progression. This blockade leads to epigenetic reprogramming by reducing histone serotonylation (H3Q5ser) at the promoters of DNA repair genes. The resulting impairment in DNA repair pathways triggers genomic instability, proteostasis imbalance, and endoplasmic reticulum stress, ultimately culminating in pyroptotic cell death.^478^ Fosinopril treatment increased reactive oxygen species (ROS) levels, leading to the activation of Bax and a subsequent downregulation of mitochondrial membrane potential (MMP). This mitochondrial dysfunction triggered the cleavage of caspase-9 and caspase-3, culminating in GSDME-mediated pyroptosis. Consequently, fosinopril suppressed tumor growth in a subcutaneous xenograft model of non-small cell lung cancer.^479^

Collectively, these findings demonstrate that targeting GSDMD with either specific inhibitors or activators represents a promising therapeutic strategy for controlling disease progression. However, current compounds lack absolute specificity for GSDMD, potentially leading to off-target effects or unexplored biological functions. Therefore, future studies should leverage AI-supported technologies to design or screen for highly specific drugs to facilitate clinical translation. Furthermore, while recent research has predominantly focused on GSDMD, the therapeutic potential of targeting other GSDMs warrants further investigation.

### Material-based delivery systems

Emerging studies have engineered various materials to deliver or activate GSDMs, notably GSDMD and GSDME, to induce pyroptosis for therapeutic applications, particularly in cancer treatment.^480–485^

Chen et al. developed iNV-GOV, a tumor-targeted viral delivery platform. This system utilizes genetically engineered, immune-compatible cell membranes expressing a chimeric antigen receptor (CAR) to cloak oncolytic viruses (OVs), thereby shielding virions from immune recognition while guiding them to tumor sites. The OV payload encodes a GSDMD-NT fragment driven by a heat-shock promoter. Upon stimulation with ultrasound-induced mild hyperthermia, the system triggers tumor-specific pyroptosis. This mechanism not only accelerates oncolysis but also promotes rapid viral release from lysed cells, amplifying infection in neighboring tumor populations. Following systemic administration, iNV-GOV demonstrated efficient tumor targeting and elicited robust antitumor immunity in patient-derived xenograft models within humanized mice.^486^ Zhang et al. developed a ROS-responsive liposomal nanoplatform (LipoNCs@pGSDMB) designed to co-deliver a polymeric STING agonist and a GSDMB expression plasmid, which enables dual neuro-immune modulation. Upon tumor-selective activation within metastatic bone niches, the nano-therapy induces STING-driven immune priming alongside GSDMB-mediated pyroptosis. In breast cancer bone metastasis models, this approach demonstrated remarkable efficacy, achieving 94% tumor suppression, complete resolution of cancer-associated pain, and efficient bone restoration.^425^ Pu et al. developed two aggregation-induced emission (AIE) pyroptosis inducers, CM1-NIC@F127 and CM2-NIC@F127, which proficiently generate reactive nitrogen species (RNS) under hypoxic tumor conditions. These agents enable the real-time visualization of key pyroptotic features, including mitochondrial swelling and the formation of large vesicles. Mechanistically, mitochondria undergo dysfunction following phototherapy due to their hypersensitivity to free radicals. This damage triggers caspase-1 activation, leading to the cleavage of GSDMD. Notably, CM1-NIC@F127 has been shown to promote dendritic cell (DC) maturation and cytotoxic T-cell activation via enhanced immunogenic cell death (ICD), resulting in the inhibition of both primary and distant tumor growth.^487^ Miao et al. developed HV-FI, a hybrid nanoplatform coated with GSDME-enriched vesicles and red blood cell membranes, to treat fibrosarcoma. By encapsulating ICG-loaded Fe_3_O_4_, the system combines photothermal therapy with pyroptosis. The delivered GSDME renders tumor cells susceptible to GZMB released by infiltrating CTLs, creating a synergistic cycle of tumor cell death and immune activation. This approach achieves complete tumor clearance and establishes long-term immunological memory against recurrence.^488^

Overall, these material-based delivery systems demonstrate significant therapeutic potential by synergistically activating pyroptosis and parallel immune pathways. Despite this promise, comprehensive safety evaluations are lacking. Future studies must prioritize assessing the biocompatibility of these materials to ensure they do not precipitate excessive inflammatory responses or systemic toxicity.

## Conclusions and perspectives

Since the identification of GSDMD as the primary executioner of pyroptosis in 2015, our understanding of GSDMs has expanded significantly. While the mechanisms of GSDM activation and pore formation have been extensively investigated, the subsequent transition from pore formation to cell rupture is not fully understood. Although NINJ1 has been identified as the mediator of this final rupture event, the precise molecular mechanism and physiological implications of NINJ1-driven plasma membrane disruption require further investigation. Moreover, while recent studies indicate that GSDMD and GSDME can be directly activated by specific PTMs, how these modifications exert functions in distinct physiological or pathological contexts remains to be fully explored. Another critical question is whether these PTMs act independently, sequentially or in a combinatorial manner on individual GSDM molecules. Systematic mapping of PTMs across diverse biological contexts, alongside functional analysis using mimetic mutants, is essential to elucidate how these PTMs modulate inflammatory outcomes. Furthermore, it is crucial to determine how different cell types modulate their membrane repair machinery and how this variability establishes the specific threshold for pyroptosis.

Accumulating evidence highlights the dual nature of pyroptosis in disease pathology. While pyroptosis is essential for host defense and tumor suppression, it can paradoxically drive pathogen dissemination, chronic inflammation, and tumor progression depending on the microenvironment. Significant gaps persist regarding the regulation of GSDMs under resting versus stressed conditions and regarding the precise mechanisms that mediate the transition of their function between beneficial and harmful outcomes. Recent studies also suggest that GSDM activation does not inevitably lead to cell death. Instead, cell fate appears to be determined by the extent, localization, and dynamics of pore formation rather than only by GSDM cleavage. For instance, in hyperactivated macrophages, limited numbers of GSDMD pores function as sublytic channels that permit mediator release while preserving cell viability. The mechanisms preventing these cells from rupturing remain unclear, although the regulation of NINJ1-mediated lysis is a potential factor.

GSDMs are also found to act as molecular switches, tipping the balance between cell death, immune signaling, and tissue homeostasis. Their activities are strictly governed by cell-type specificity, proteolytic cleavage, and the subcellular localization of the resulting fragments. Despite significant progress, the regulatory mechanisms that allow GSDMs to function so differently across diverse biological contexts remain to be elucidated. With the development of real-time visualization technologies, it is now possible to distinguish the spatiotemporal dynamics that differentiate physiological functions from death induction. However, significant questions remain regarding the factors that dictate the subcellular localization of GSDM fragments to organelles such as mitochondria, the endoplasmic reticulum, and the nucleus. While electrostatic interactions between GSDM-NT and negatively charged phospholipids explain plasma membrane targeting, the determinants of organelle specificity are unclear. Although differences in lipid composition may bias binding, quantitative data on affinity and selectivity are lacking. Moreover, it remains unknown whether accessory proteins or ionic environments guide this targeting. It is also possible that PTMs induce subtle conformational changes, exposing specific motifs that favor distinct subcellular destinations. Future studies integrating structural biology, lipid omics, and live-cell imaging will be critical to delineate these mechanisms and understand how localization shapes functional outcomes.

The therapeutic landscape is evolving rapidly to address these complexities, with current strategies exploring specific small-molecule inhibitors such as NSA and DSF, as well as novel agonists to modulate GSDM activity. However, given the potential for off-target effects and the dual nature of GSDMs, the development of precision therapies remains a critical challenge. Future translational efforts must leverage artificial intelligence for drug screening and utilize advanced material-based delivery systems to achieve context-specific modulation. Moreover, as most existing studies are currently confined to cellular and murine models, a concerted effort is required to bridge the gap between preclinical findings and clinical translation.
